# Astrocytes contribute to toll-like receptor 2-mediated neurodegeneration and alpha-synuclein pathology in a human midbrain Parkinson’s model

**DOI:** 10.1186/s40035-024-00448-3

**Published:** 2024-12-16

**Authors:** Fiona Weiss, Laura Hughes, Yuhong Fu, Cedric Bardy, Glenda M. Halliday, Nicolas Dzamko

**Affiliations:** 1 School of Medical Sciences, Faculty of Medicine and Health and the Brain and Mind Centre, University of Sydney, Camperdown, NSW 2050 Australia; 2 Laboratory for Human Neurophysiology and Genetics, South Australian Health and Medical Research Institute (SAHMRI), Adelaide, SA Australia

**Keywords:** Parkinson’s disease, Toll-like receptor 2, Alpha-synuclein, Astrocyte, Lysosome

## Abstract

**Background:**

Parkinson’s disease (PD) is characterised by degeneration of ventral midbrain dopaminergic (DA) neurons and abnormal deposition of α-synuclein (α-syn) in neurons. Activation of the innate immune pathogen recognition receptor toll-like receptor 2 (TLR2) is associated with exacerbation of α-syn pathology. TLR2 is increased on neurons in the PD brain, and its activation results in the accumulation and propagation of α-syn through autophagy inhibition in neurons. In addition to the aggregation and propagation of pathological α-syn, dysfunction of astrocytes may contribute to DA neuronal death and subsequent clinical progression of PD. However, the role of astrocytes in TLR2-mediated PD pathology is less explored but important to address, given that TLR2 is a potential therapeutic target for PD.

**Methods:**

Induced pluripotent stem cells from three controls and three PD patients were differentiated into a midbrain model comprised of neurons (including DA neurons) and astrocytes. Cells were treated with or without the TLR2 agonist Pam3CSK4, and α-syn pathology was seeded using pre-formed fibrils. Confocal imaging was used to assess lysosomal function and α-syn pathology in the different cell types, as well as DA neuron health and astrocyte activation.

**Results:**

TLR2 activation acutely impaired the autophagy lysosomal pathway, and potentiated α-syn pathology seeded by pre-formed fibrils in PD neurons and astrocytes, leading to degeneration and loss of DA neurons. The astrocytes displayed impaired chaperone-mediated autophagy reducing their ability to clear accumulated α-syn, and increases of A1 neurotoxic phenotypic proteins SerpinG1, complement C3, PSMB8 and GBP2. Moreover, the phenotypic changes in astrocytes correlated with a specific loss of DA neurons.

**Conclusions:**

Taken together, these results support a role for astrocyte dysfunction in α-syn accumulation and DA neuronal loss following TLR2 activation in PD.

**Supplementary Information:**

The online version contains supplementary material available at 10.1186/s40035-024-00448-3.

## Background

Parkinson’s disease (PD) is a progressive neurodegenerative disorder characterised by the loss of substantia nigra pars compacta dopaminergic (DA) neurons and the presence of proteinaceous inclusions termed Lewy bodies (LBs) and Lewy neurites [1]. The progression of PD involves the appearance of LBs in affected brain regions, which spread in a time-dependent manner commencing prior to the loss of DA neurons [1]. The major component of LBs is the protein α-synuclein (α-syn) [2, 3]. Subsequently the development of α-syn pathology has been a focal point in PD research, with fibrillar forms utilised to demonstrate α-syn aggregation and propagation in cells and animal models of PD [4–7].

One model of α-syn pathology development and propagation involves activation of toll-like receptor 2 (TLR2). TLRs are activated by the recognition of pathogen-associated molecular patterns, derived from non-self signals such as bacteria or viral particles, as well as self-originating damage-associated molecular patterns that result from tissue damage or cellular stress. TLR2 is increased on neurons in the PD brain and associated with increased levels of α-syn pathology [8]. In addition, activation of TLR2 on neurons inhibits autophagy, leading to an increase of α-syn levels and potentiation of pre-formed fibril (PFF)-mediated α-syn pathology [9]. Moreover, inhibition of TLR2 with small molecules or neutralising antibodies can ameliorate the development of α-syn pathology in vitro [9, 10]. In vivo, neutralising antibodies or genetic deletion of TLR2 results in a reduction of TLR2-mediated α-syn pathology and rescues the loss of DA neurons [10–12]. These studies collectively highlight TLR2 as a potential disease-modifying target for PD.

Previous studies have predominantly focused on the development of neuronal α-syn pathology, DA degeneration and death. However, the contribution of astrocytes to PD pathogenesis is increasingly being recognised. Analysis of postmortem brain tissues has shown evidence of astrocytic accumulation of α-syn in PD [13–15], and that overexpression of mutant α-syn in mice leads to disruption of normal astrocytic functions such as blood–brain barrier control, resulting in a significant loss of DA neurons [16]. Additionally, PD mutant astrocytes displaying dysfunction through abnormal α-syn accumulation and altered autophagy have been directly implicated in the loss of DA neurons in an induced pluripotent stem cell (iPSC)-derived co-culture model [17]. Astrocytes with α-syn inclusions are observed in regions without LB deposition but close to terminal axons [13], supporting the hypothesis of α-syn spread through neuron-astrocyte interactions. Moreover, neuron-derived α-syn can activate TLR2 on astrocytes, enhancing the production of pro-inflammatory cytokines such as TNF-α and IL-1β [18]. Astrocytes in PD postmortem brain samples and α-syn-overexpressing rodents show increased TLR2 expression, and inhibition of TLR2 activation significantly reduces astroglial α-syn accumulation and the associated pro-inflammatory response [11].

A shift to the pro-inflammatory A1 phenotype in astrocytes may also contribute to neuronal loss in PD [19]. Transcriptome analysis has suggested astrocytic shift between the proinflammatory A1 phenotype and the neuroprotective A2 phenotype in a stimulus-dependent manner [19, 20]. An increase in A1 astrocytes expressing complement C3 has been observed in PD brains, and treatment of astrocytes in vitro with cytokines TNF-α, IL-1α and C1q resulted in a shift to A1 astrocytes, leading to a loss of their ability to maintain neuronal survival [19]. Glial-derived neurotrophic factors promote the survival and differentiation of DA neurons [21]. Therefore, the loss of normal supportive functions can be detrimental to DA neurons specifically. In a rodent model of PD, the aggregation of α-syn induced by α-syn PFFs resulted in a decreased number of DA neurons and an increased number of complement C3-positive astrocytes in the midbrain, which were rescued by inhibition of the conversion of astrocytes to an A1 pro-inflammatory phenotype [22]. Moreover, treatment of astrocytes in vitro with α-syn PFFs induced upregulation of A1-associated transcripts such as SerpinG1, and treatment of neurons with the A1 astrocyte-conditioned medium induced neuronal death, which was rescued with prevention of A1 astrocytic conversion [23]. Therefore, a role for astrocytic dysfunction and pro-inflammatory phenotype in DA neuronal loss in PD is emerging.

In this study, stem cell-derived midbrain cultures comprising astrocytes, MAP2-positive neurons, and TH-positive DA neurons from three controls and three PD patients were employed to determine how astrocytes contribute to the TLR2-mediated α-syn pathology in a human cell model of PD. The study outcomes support a role for astrocytic dysfunction in the loss of DA neurons in PD following activation of TLR2.

## Materials and methods

### Preparation of α-syn PFFs

Human recombinant α-syn monomeric protein at 12 mg/ml in 10 mM Tris and 50 mM NaCl, pH 7.6 was purchased from Proteos (Kalamazoo, MI), and PFFs were prepared as recommended [24]. The endotoxin levels of the recombinant monomer are reported as < 0.05 EU/mg. Briefly, monomeric protein was diluted to 5 mg/ml in sterile Dulbecco’s phosphate-buffered saline (dPBS) (Ca^2+^- and Mg^2+^-free; Thermo Fisher Scientific, Waltham, MA) and continuously shaken at 1000 rpm on an orbital shaker (Thermomixer C, Eppendorf, Hamburg) that was placed in a 37 °C incubator for 7 days. The presence of amyloid fibrils was confirmed by thioflavin T assay. The resulting fibrillar α-syn was stored at − 80 °C in aliquots of 25 μl. Immediately before treating the cells, the α-syn PFFs were thawed at room temperature, diluted in sterile dPBS to 0.1 mg/ml and sonicated for 2 min at 40% amplitude with 1 s on/off pulse durations (Sonicator Q125, QSonica, Newtown, CT) to obtain a homogenous PFF suspension for experiments as previously characterised [25].

### iPSC line

iPSCs from two PD patients and one control were obtained from the Golub Capital iPSC Parkinson’s Progression Marker Initiative (PPMI) sub-study (www.ppmi-info.org/cell-lines). The investigators within PPMI contributed to the design and implementation of PPMI and/or provided data and collected samples but did not participate in the analysis or writing of this report. For up-to-date information on the PPMI study, please visit www.ppmi-info.org. These lines were differentiated to neural stem cells (NSCs) as described previously [26]. The third PD line and two additional control lines were generated in house by reprogramming fibroblasts obtained from the Coriell cell repository (Camden, NJ). The generation and characterisation of these lines has been described previously [9]. Available demographic data associated with all cell lines used are shown in Table 1. NSCs were differentiated from iPSCs using the PSC neural induction kit (Thermo Fisher Scientific), and then cultured and maintained as described previously [8, 9, 25, 27]. All work with human iPSC was approved by the University of Sydney Human Research Ethics Committee (2017/094).

**Table 1:** Demographic data of iPSC cell lines used in this study

Cell line No.	Type	Age	Sex
7428	Ctrl Line 1	72	F
#ND35044	Ctrl Line 2	77	M
#ND38530	Ctrl Line 3	55	M
#ND29494	PD Line 1	60	M
15733	PD Line 2	80	M
10106	PD Line 3	61	F

iPS cells obtained from the Coriell cell repository were indicated with # with the catalogue numbers provided

### Midbrain differentiation from NSCs

NSCs were differentiated into a midbrain model following the protocol of Zabolocki and colleagues [28]. Briefly, NSCs were split from a 70%–80% confluent well at 1:4–1:5 ratio (cell line-dependent) and seeded on tissue culture plates coated with Geltrex diluted 1:100 in Advanced DMEM (ThermoFisher) for 1 h at 37 °C prior to plating cells. NSCs were then cultured in neural expansion medium composed of 50% Advanced DMEM, 50% Neurobasal, 1 × neural induction supplement and 1 × penicillin–streptomycin (all from Thermo Fisher Scientific) until cells were ~ 40%–50% confluent. Medium was then changed to Midbrain neural progenitor medium, which was composed of DMEM/F12 + GlutaMAX (Thermo Fisher Scientific) supplemented with 1 × SM1 supplement (STEMCELL Technologies, Vancouver, BC), 1 × N2-A supplement (STEMCELL Technologies), 200 ng/ml Sonic Hedgehog (SHH) (PeproTech, Cranbury, NJ), 100 ng/ml fibroblast growth factor-8 (FGF8b) (PeproTech, Cranbury, NJ), 200 nM ascorbic acid (L-AA) (Sigma), 1 μg/ml laminin (Thermo Fisher Scientific) and 1 × penicillin–streptomycin (Thermo Fisher Scientific). The midbrain neural progenitor cells (mNPCs) were maintained at a high density (100% confluency by day 4) in the midbrain neural progenitor medium with half medium changed every 2 days for 8 days. For further maturation, the midbrain progenitor cells were then dissociated using Accutase (STEMCELL Technologies) and seeded at a density of 8.1 × 10^4^–1 × 10^5^ cells/cm^2^ (cell line-dependent) onto tissue culture treated plates coated with 20 μg/ml poly-l-ornithine (Sigma-Aldrich, St. Louis, MO) and 10 μg/ml laminin diluted in midbrain progenitor medium. The following day, the medium was changed to midbrain maturation medium composed of BrainPhys™ (STEMCELL Technologies,Vancouver, BC) supplemented with 1 × SM1 (STEMCELL Technologies), 1 × N2A (STEMCELL Technologies), 20 ng/ml glial-derived neurotrophic factor (GDNF) (Peprotech), 20 ng/ml brain-derived neurotrophic factor (BDNF) (Peprotech, Cranbury, NJ), 0.5 mM dibutyryl cyclic-AMP (db-cAMP) (STEMCELL Technologies), 200 nM L-AA (Sigma-Aldrich), 1 μg/ml laminin and 1 × penicillin–streptomycin. Half medium was changed every 2–3 days with fresh midbrain maturation medium. After 14 days, the concentration of growth factors was halved to 10 μg/ml BDNF, 10 μg/ml GDNF and 0.25 mM db-cAMP. Cells were then treated with 1 μg/ml TLR2 agonist Pam3CSK4 (InvivoGen, San Diego, CA) and/or 1 μg/ml α-syn PFFs diluted in neuronal maturation medium. The cells were then fixed or lysed at the time points indicated. For autophagy flux measures, midbrain cells were treated with 200 nM Bafilomycin A1 (Sigma-Aldrich) for 4 h. Bafilomycin A1 was diluted in midbrain medium and added with half medium change.

### Immunoblot analysis

For immunoblot analysis, cells were lysed in buffer containing 50 mM Tris–HCl pH 7.5, 1 mM ethylene glycol tetraacetic acid, 1 mM ethylenediamne tetraacetic acid (EDTA), 1 mM sodium orthovandate, 50 mM sodium fluoride, 5 mM sodium pyrophosphate, 0.2 M sucrose, 1 mM benzamidine, 1 mM phenylmethylsulphonyl fluoride (PMSF) and 1% (*v*/*v*) Triton X-100. Samples were snap frozen in liquid nitrogen and stored at − 80 °C until use. Thawed lysates were then clarified by centrifugation at 12,000 × *g* for 20 min at 4 °C and protein concentrations measured using a bicinchoninic acid (BCA) assay (Pierce BCA Protein Assay Kit, Thermo Fisher Scientific) according to the manufacturer’s instructions. Samples were made up in 1 × NuPAGE lithium dodecyl sulfate (LDS) buffer (Thermo Fisher Scientific) with 5% β-merceptoethanol added. Up to 30 μg of protein lysate was heated at 70 °C for 10 min, separated by reducing 4%–12% Novex Tri-glycine gels (Thermo Fisher Scientific) and transferred onto nitrocellulose membranes (Bio-Rad, Hercules, CA). Membranes were blocked with 5% skim milk dissolved in Tris-buffered saline with 0.1% Tween-20 (TBST). Membranes were then cut based on molecular weight markers and incubated overnight at 4 °C with primary antibodies (Table S1) diluted at 1:1000 in 5% skim milk in TBST. Membranes were washed three times for 5 min each in TBST and then incubated with anti-mouse or anti-rabbit horseradish peroxidase secondary antibody (Bio-Rad, 1:5000 dilution in 2.5% skim milk in TBST) for 2 h at room temperature. Enhanced chemiluminescence reagent (GE Healthcare, Chicago, IL) and a Chemidoc MP digital imaging system (Bio-Rad) were used for detection. β-Actin was used as a loading control.

To re-probe blots with additional primary antibodies, membranes were washed for 10 min then incubated in stripping buffer (0.2 M glycine, 0.1% SDS *w*/*v* and 1% Tween-20 in distilled H_2_O, pH adjusted to 2.2 with 2 M HCl) on a rocking platform for 7 min. Then the buffer was replaced, and the membranes were further incubated for 5 min, washed twice with 1 × PBS and then once with 1 × TBST. Before re-probing for other antibodies, membranes were blocked with 5% skim milk in TBST for 1 h on a rocking platform, then incubated with a primary antibody at 4 °C overnight and developed as previously described. The relative levels of each protein of interest were analysed using the Fiji ImageJ software (NIH, Bethesda, MA). The intensity of each protein band was quantified and expressed as arbitrary units normalised to β-actin.

### Flow cytometry

Cells were detached with Accutase (STEMCELL Technologies) and strained through a 100-μm cell strainer (Falcon, Corning, NY). Cells were then washed with 1 × PBS, pelleted by centrifugation (300 × *g* for 5 min at 4 °C), resuspended in 1 × PBS containing FcR Blocking Reagent (Miltenyi Biotec, Bergisch Gladbach, Germany), Live/Dead stain 780 (Thermo Fisher Scientific) and PE-conjugated anti-TLR2 antibody (BioLegend, San Diego, CA), and incubated for 30 min at 4 °C with rocking in the dark. Cells were then centrifuged at 300 × *g* for 5 min at 4 °C, washed twice with fluorescence-activated cell sorting (FACS) buffer containing 1 × PBS, 1 mM EDTA, 25 mM HEPES and 1% heat-inactivated fetal bovine serum (HI-FBS), pH 7.4, and fixed with 4% paraformaldehyde (PFA) at room temperature in the dark for 10 min. Cells were again centrifuged at 300 × *g* for 5 min at 4 °C, washed twice with FACS buffer and resuspended in permeabilisation/blocking buffer made from 10 × Perm/Wash buffer (BD Biosciences) diluted to 1 × in sterile distilled water with 5% FBS added containing primary antibodies anti-MAP2 (Thermo Fisher Scientific), anti-GFAP (Thermo Fisher Scientific) and anti-TH (Thermo Fisher Scientific) and incubated for 2 h at 4 °C with rocking in the dark (Antibody details in Table S1). Cells were then centrifuged at 300 × *g* for 5 min at 4 °C, washed twice with permeabilisation/blocking buffer and then resuspended in permeabilisation/blocking buffer containing secondary antibodies donkey anti-rabbit 405 (Abcam, Cambridge, UK), donkey anti-rat AF488 (Thermo Fisher Scientific) and donkey anti-chicken AF647 (Jackson ImmunoResearch, West Grove, PA) and incubated for 1 h at 4 °C with rocking in the dark. Cells were centrifuged at 300 × *g* for 5 min at 4 °C, washed once in permeabilisation/blocking buffer and twice in FACS buffer before being resuspended in 300 μl FACS buffer and transferred to flow cytometry tubes (Falcon) through a 40-μm cell strainer cap and kept on ice until acquisition on a Cytek Aurora spectral analyser (Cytek Biosciences, Fremont, CA). A minimum of 200,000 events were recorded per sample and data analysed using FlowJo Version 10.7.1 (BD, Franklin Lakes, NJ).

### Immunocytochemistry

For immunocytochemistry, cells were seeded in 96-well plates (PhenoPlates, PerkinElmer, Waltham, MA) pre-coated with 20 μg/ml poly-l-ornithine (Sigma-Aldrich) and 10 μg/ml laminin (Thermo Fisher Scientific) at a density of 8.1 × 10^4^–1 × 10^5^ cells/cm^2^ (cell line-dependent) in midbrain maturation medium and fixed in 4% PFA for 15 min at room temperature. After fixation, wells were washed twice with 1 × PBS and permeabilised with 0.3% Triton X-100 for 15 min at room temperature. Cells were then blocked in 3% BSA for 1 h at room temperature and incubated with primary antibodies (Table S1) diluted in 3% BSA for 3 h at room temperature. Cells were then washed three times in 1 × PBS and incubated with AlexaFluor secondary antibodies (all used at 1:300, Thermo Fisher Scientific) diluted in 3% BSA for 1 h at room temperature protected from light. After incubation, cells were then washed three times with 1 × PBS with DAPI added to the last wash for 10 min at a dilution of 1:10,000 (Sigma-Aldrich). To stain cells with Lysotracker, differentiated and treated cells were incubated with Lysotracker Deep Red (Thermo Fisher Scientific) for 1 h at 37 °C before fixation. Cells were then blocked and permeabilised with 0.1% saponin (Sigma-Aldrich) and 3% BSA diluted in PBS for 1 h at room temperature, and primary and secondary antibodies were diluted in blocking buffer and stained as previously described. All cells were left in 1 × PBS with 0.02% sodium azide (Sigma) added to the wells to prevent contamination and stored at 4 °C until imaging. Images were captured on an inverted confocal microscope (A1R, Nikon, Tokyo, Japan) using NIS elements AR software (Nikon, Tokyo, Japan) with optical configuration settings kept consistent within each experiment across lines.

### Image analysis

Confocal images for α-syn and pS129 α-syn were obtained at 40 × objective magnification, whereas images for autophagy markers P62 and LAMP2A and Lysotracker were captured at 60 × objective magnification. Images were saved as .nd2 file format and imported into Fiji ImageJ for analysis. For all image analysis the threshold tool was used to highlight stained areas in the field. To analyse signal intensity the integrated density was measured, and to measure the number and/or size of α-syn and pS129 α-syn aggregates, particles over 0.5 μm^2^ for α-syn and 1 μm^2^ for pS129 α-syn were measured through the analyse particles tool. To measure signal in respective cell populations the structural marker was used to draw a ROI of the cell and the stain of interest measured within the ROI. Cell number was counted for each image based on DAPI staining. Cells touching the edges of the image were excluded from the final analysis. Six to eight images were analysed for each treatment per cell line. Fluorescence integrated density and particle number were normalised to the cell number in the field. For lysosomal number analysis, the threshold tool was used to highlight lysotracker-labelled lysosomes in S100β-positive cells, and the analyse particles tool used to assess the number of lysosomes per cell. Lysosomes over 2 μm^2^ were considered enlarged and the number of S100β-positive cells containing enlarged lysosomes was counted. For LAMP2A positioning analysis, LAMP2A puncta within 5 μm of the nucleus was considered perinuclear, and LAMP2A puncta inside and then outside of this ROI was measured.

### Lactate dehydrogenase (LDH) release assay

LDH was measured in the tissue culture medium using the CyTox96 assay kit (Promega, Madison, WI) as per the manufacturer's instructions. Medium was removed from cells at the indicated times of fixation and stored at − 80 °C until use. The CytoTox96 Reagent was prepared by mixing 12 ml of Assay buffer with the Substrate Mix immediately before use. Fifty microliters of culture medium was added in triplicate to a 96-well transparent plate (Greiner Bio-One, Frickenhausen, Germany) and mixed with the CytoTox96 Reagent at a 1:1 ratio. The samples were then incubated for 30 min at room temperature protected from light. At the end of the incubation, 50 μl of Stop Solution was added into each well. The absorbance was measured at 492 nm and read within one hour after the addition of the Stop Solution using a Tecan plate reader. Three wells containing medium only (without cells) were used to determine background readings for each plate and their mean value subtracted from the absorbance reading of each well to calculate the final reading.

### Statistical analysis

Statistical analysis and graph creation were performed using Prism (Graphpad Software, Boston, MA). Data were analysed with one-way or two-way ANOVA and Dunnet’s post-hoc test. For all analyses, the treatment groups were compared to the control group. Pearson correlation analysis was used to determine associations between two variables, with *r* values indicating the strength of linear relationship between two variables. For all analyses, significance was accepted at* P* < 0.05 and bar graphs are shown as mean ± SEM.

## Results

### Characterisation of NSC-derived midbrain model

**Fig. 1:**
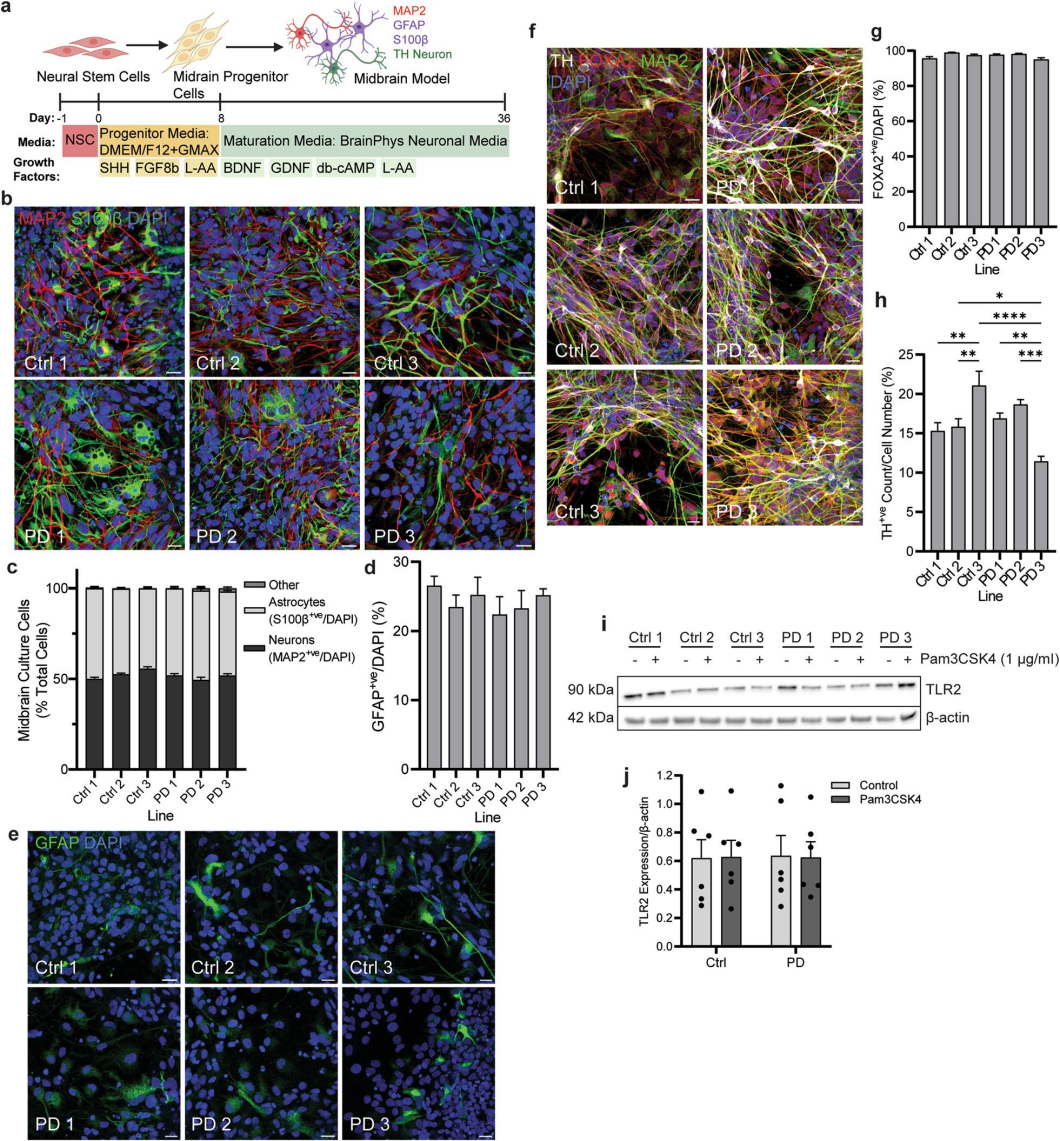
Generation and characterisation of midbrain culture from six stem cell PD patient lines. **a** Diagram of differentiation protocol as published by Zabolocki and colleagues [28]. **b** Differentiated cells were fixed and stained for astrocyte and neuronal markers S100β (green) and MAP2 (red) respectively. Confocal images taken at 40 × magnification. Scale bar, 20 μm. **c** Graph shows the numbers of astrocytes and neurons in culture expressed as percentage of total cells, displayed as mean ± SEM. Cells negative for either stain were classified as “other”. **d** Graph shows the number of GFAP positive astrocytes in culture, expressed as percentage of total cells, displayed as mean ± SEM. **e** differentiated cells were fixed and stained for astrocyte marker GFAP (green). Confocal images taken at 40 × magnification, scale bar, 20 μm. **f** Midbrain cells were fixed and stained for DA neuronal markers TH (grey) and FOXA2 (red), and general neuronal marker MAP2 (green). Confocal images taken at 40 × magnification, scale bar, 20 μm. **g**, **h** Graphs show the number of FOXA2- and TH-positive cells in culture, respectively, expressed as percentage of the total cells, displayed as mean ± SEM. **i** Midbrain cells were treated with TLR2 agonist Pam3CSK4 for 24 h and lysed for immunoblot detection of TLR2 with β-actin used as a loading control. **j** Graph shows quantification of TLR2 expression normalised to β-actin following Pam3CSK4 treatment, displayed as mean ± SEM. For all graphs *P < 0.05, **P < 0.01, ***P < 0.001, ****P < 0.0001

Midbrain cells were differentiated from NSCs based on the protocol of Zabolocki and colleagues [28] (Fig. 1a). Immunocytochemistry analysis of the differentiated cells showed robust expression of the neuronal marker MAP2 and the astrocyte marker S100β (Fig. 1b). There was no significant difference in MAP2 or S100β expression between the six cell lines, with astrocytes accounting for 40%–45% of total cells, and neurons accounting for the other 55%–60% of the total cells depending on the cell line (Fig. 1c). Notably, GFAP varied more as an astrocyte marker, with GFAP-positive cells comprising 20%–25% of the total midbrain cells, but this was also not significantly different between the lines (Fig. 1d, e). The expression of FOXA2 identified these cells as floor-plate derived [29, 30], with above 85% of the total midbrain cells positive for this marker across all lines (Fig. 1f, g). Additionally, the expression of tyrosine hydroxylase (TH) in 10%–20% of cells established a population committed to DA neuron fate (Fig. 1f, h). The percentage of TH-positive neurons significantly differed between lines. Notably, Ctrl line 3 displayed the highest percentage of TH-positive cells with on average 20% of the total cells, and PD line 3 showed the lowest with an average of 10% of the total cell count (Fig. 1h). Immunoblot analysis showed that the expression of TLR2 did not differ significantly across the six cell lines following 24-h treatment with the TLR2 agonist Pam3CSK4 (Fig. 1i, j).

### Response of control and PD midbrain cells to TLR2 activation

**Fig. 2:**
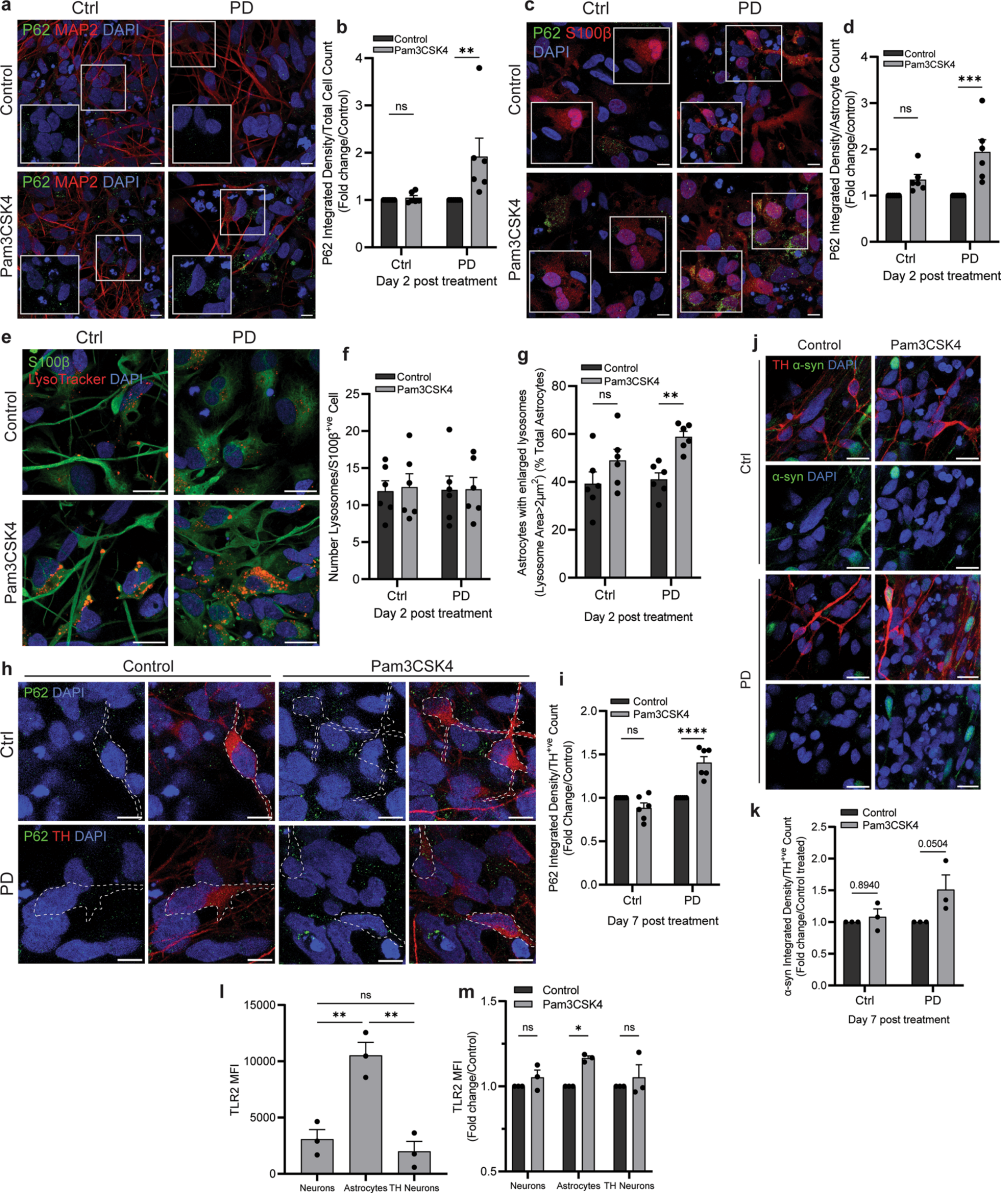
Response of control and PD midbrain cells following TLR2 activation. Midbrain cells were treated with 1 μg/ml Pam3CSK4 for 2 and 7 days as indicated. **a** Cells were stained for P62 (green) and MAP2 (red). Confocal images were taken at 60 × magnification with 8 images captured per condition. Scale bar, 10 μm. **b** Graph shows fold change in P62 intensity/cell normalised to the control, displayed as mean ± SEM  (*n *= 6). **c** Cells were stained for P62 (green) and S100β (red). Confocal images were taken at 60 × magnification with 8 images captured per condition.Scale bar, 10 μm. **d** Graph shows fold change in P62 intensity/astrocyte normalised to the control, displayed as mean ± SEM (*n *= 6). **e** Cells were stained with Lysotracker (red) for 1 h at 37 °C prior to fixation and co-stained for S100β (green). Confocal images taken at 60 × magnification with 8 images captured per condition. Scale bar, 10 μm. **f**, **g** Graphs show the average number of lysosomes/astrocyte and the average percentage of astrocytes with enlarged lysosomes (> 2 μm^2^) ± SEM (*n *= 6). **h** Cells were stained for P62 (green) and TH (red). Confocal images were taken at 60 × magnification with 8 images captured per condition. Scale bar, 10 μm. **i** Graph shows fold change in P62 intensity/TH neuron normalised to the control, displayed mean ± SEM (*n *= 6). **j** Cells were stained for α-syn (green) and TH (red). Confocal images were taken at 40 × magnification with 6 images captured per condition. Scale bar, 20 μm. **k** Graph shows fold change in α-syn integrated density/TH neuron normalised to the control, displayed as mean ± SEM (*n *= 3). **l**, **m** Graphs show flow cytometry analysis of TLR2 in the different cell populations of PD midbrain cells, and the fold change in TLR2 following 24 h Pam3CSK4 treatment, respectfully, displayed as mean ± SEM (*n *= 3). For all graphs *P < 0.05, **P < 0.01 ***P < 0.001, ****P < 0.0001, ns = not significant

Control and PD lines were then treated with Pam3CSK4 for two and seven days. The two-day samples were used to assess lysosomal function in response to TLR2 activation and revealed an increase in P62 only in PD samples whether looking at all cells (Fig. 2a, b) or astrocytes (Fig. 2c, d). On day 2, the lysosome number did not increase in astrocytes in control or PD lines following Pam3CSK4 treatment (Fig. 2e, f), but in PD samples the percentage of astrocytes with enlarged lysosomes (> 2 μm^2^) was significantly increased (Fig. 2g). On day 7 following TLR2 activation, accumulation of P62 (Fig. 2h, i) and α-syn (Fig. 2j, k) was observed in TH neurons only in PD samples. To further characterise the PD lines, the expression of P62 and α-syn was also assessed by immunoblot, with PD1 having significantly less α-syn protein than PD2 and PD3, while the expression of P62 was similar across all PD lines (Fig. S1). As the immunoblot data contained mixed populations, flow cytometry was used to further investigate differences in TLR2 expression between the cell populations in PD midbrain cells (gating strategy is shown in Fig. S2). The results showed that the expression of TLR2 was higher in GFAP-positive astrocytes compared to MAP2-positive and TH-positive neurons (Fig. 2l). Astrocytes also responded to the TLR2 agonist Pam3CSK4 with a significant increase in TLR2 protein observed after 24 h, which was not observed in MAP2- or TH-positive neurons at this time point (Fig. 2m).

### TLR2 activation potentiates α-syn fibril-seeded pathology in a midbrain model

**Fig. 3:**
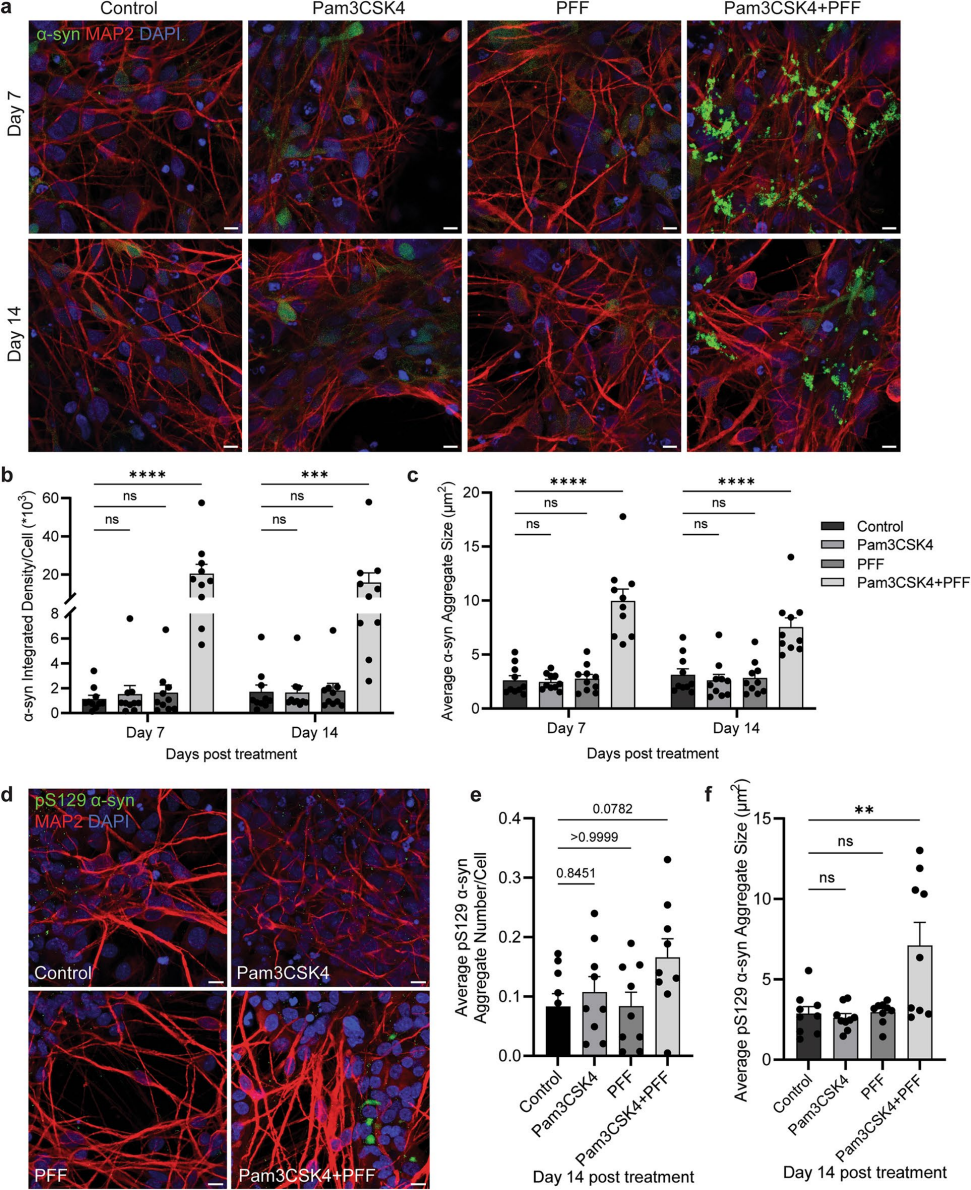
TLR2 activation potentiates α-syn fibril-seeded pathology in PD midbrain cells. Stem cell-derived midbrain model from PD patients was treated for 7 and 14 days with 1 μg/ml Pam3CSK4 and/or 1 μg/ml PFFs, or medium only as the control. **a** Cells were fixed at days indicated and stained for total α-syn (green) and MAP2 (red). Confocal images were taken at 40 × magnification with 6 images captured per condition and used for the analysis of α-syn signal intensity and particle analysis. Scale bar, 10 μm. **b** Graph shows α-syn intensity/cell number as mean ± SEM (*n* = 10). **c** Graph shows α-syn aggregate size (μm^2^) as mean ± SEM (*n* = 10). **d** Cells were fixed at day 14 and stained for pS129 α-syn (*green*) and MAP2 (*red*). Confocal images were taken at 40 × magnification with 6 images captured per condition and used for the analysis of pS129 α-syn aggregate count and size. Scale bar, 10 μm. **e** Graph shows pS129 α-syn aggregate number normalised to cell number as mean ± SEM (*n* = 9). **f** Graph shows pS129 α-syn aggregate size (μm^2^) as mean ± SEM (*n *= 9). For all graphs *P < 0.05, **P < 0.01, ***P < 0.001, ****P < 0.0001, ns = not significant

Previously it has been shown that neuronal TLR2 activation is associated with the accumulation of α-syn [8, 10], and that TLR2 activation can potentiate α-syn pathology following seeding with low levels of PFFs in SH-SY5Y cells [9]. To establish this effect in a PD midbrain model, differentiated cells were treated with 1 μg/ml of TLR2 agonist Pam3CSK4 and/or 1 μg/ml α-syn PFFs, and α-syn pathology markers measured on days 7 and 14 of treatment. Previous work has shown that the effect of TLR2 activation to potentiate α-syn pathology is large, requiring lower concentrations of PFF to avoid saturation of signal [9]. Therefore 1 μg/ml of α-syn PFFs was employed in this study rather than the more commonly used concentration of 5 μg/ml. Following dual stimulation with Pam3CSK4 and PFF, the level of α-syn and the size of α-syn aggregates were significantly increased when compared to the untreated cells on days 7 and 14 (Fig. 3a–c). Phosphorylated α-syn Ser-129 (pS129 α-syn) is a major component of LBs and may contribute to α-syn aggregation [31–33], and therefore was measured in this model to show pathology development. Increases in pS129 α-syn aggregate number and size were seen only on day 14 of treatment with Pam3CSK4 plus PFF compared to the control, but not with either Pam3CSK4 or PFF treatment alone (Fig. 3d–f).

### TLR2 activation potentiates α-syn fibril-seeded pathology in DA neurons and astrocytes

**Fig. 4:**
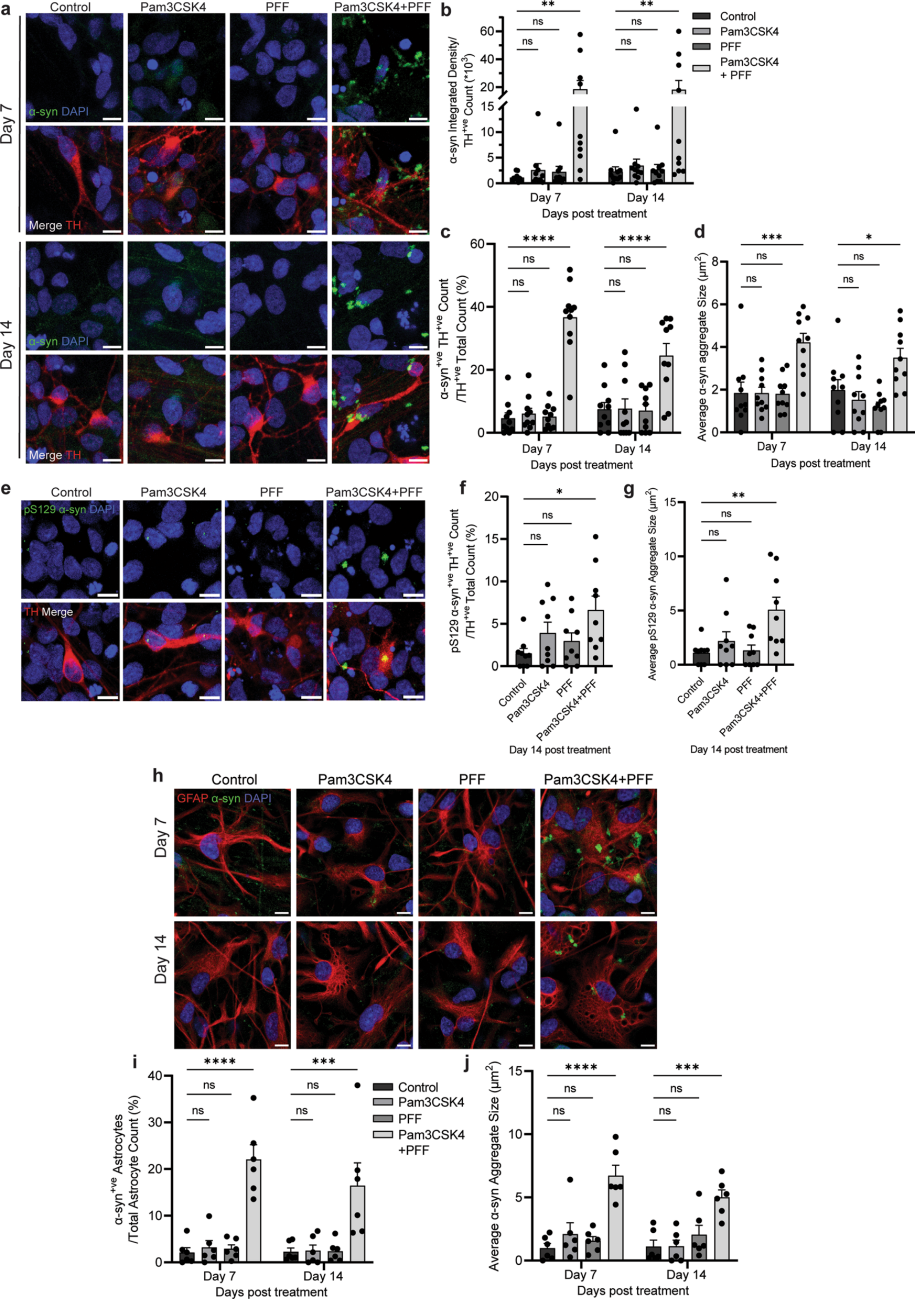
TLR2 activation potentiates α-syn PFF mediated pathology in DA neurons and astrocytes. Differentiated PD patient iPSCs were cultured for 7 and 14 days with or without 1 μg/ml Pam3CSK4 and/or 1 μg/ml PFFs. **a** Cells were fixed at the indicated timepoints and stained for total α-syn (green) and TH (red). Confocal images were taken at 40 × magnification with 6 images captured per condition and used for the analysis of α-syn signal intensity and particle analysis. Scale bar, 10 μm. **b** Graph shows α-syn integrated density/TH-positive cell number as mean ± SEM (*n* = 10). **c** Graph shows the average percentage of TH-positive cells with α-syn aggregates ± SEM (*n* = 10). **d** Graph shows α-syn aggregate size (μm^2^) measured in TH positive neurons displayed as mean ± SEM (*n* = 10). **e** Cells were fixed at day 14 post treatment and stained for pS129 α-syn (green) and TH (red). Confocal images were taken at 40 × magnification with 6 images captured per condition and used for the analysis of pS129 α-syn aggregate count and particle analysis in TH positive neurons. Scale bar, 10 μm. **f** Graph shows the average number of TH positive neurons with pS129 α-syn aggregates ± SEM  (*n *= 9). **g** Graph shows pS129 α-syn aggregate size (μm^2^) as mean ± SEM (*n* = 9). **h** Cells were fixed at the indicated timepoints and stained for total α-syn (green) and GFAP (red). Confocal images were taken at 40 × magnification with 6 images captured per condition and used for the analysis of α-syn aggregate count and particle analysis. Scale bar, 10 μm. **i** Graph shows average percentage of GFAP positive cells with α-syn aggregates ± SEM (*n* = 6). **j** Graph shows α-syn aggregate size (μm^2^) in astrocytes as mean ± SEM (*n* = 6). For all graphs *P < 0.05, **P < 0.01, ***P < 0.001, ****P < 0.0001, ns = not significant

As the degeneration of DA neurons is a hallmark of PD progression, an advantage of utilising a midbrain model including DA neurons allows investigation into pathology development in this neuronal population. DA neurons displayed significant increases of α-syn levels and aggregate size on days 7 and 14 of treatment with Pam3CSK4 plus PFF (Fig. 4a–d), and a significant increase in the number and size of pS129 α-syn aggregates on day 14 with the dual treatment of Pam3CSK4 and PFF (Fig. 4e–g). Astrocytes in PD also display abnormal accumulation of α-syn [13–15]. Consistently, with the combined treatment with Pam3CSK4 plus PFF, there were significant increases in the percentage of α-syn-positive astrocytes and the size of α-syn aggregates in astrocytes on day 7 of treatment, and the increases remained on day 14 (Fig. 4h–j). Therefore, the accumulation of α-syn with the dual treatment with Pam3CSK4 plus PFFs was not limited to neuronal cells in this midbrain model.

### TLR2 activation inhibits autophagy in a midbrain model

**Fig. 5:**
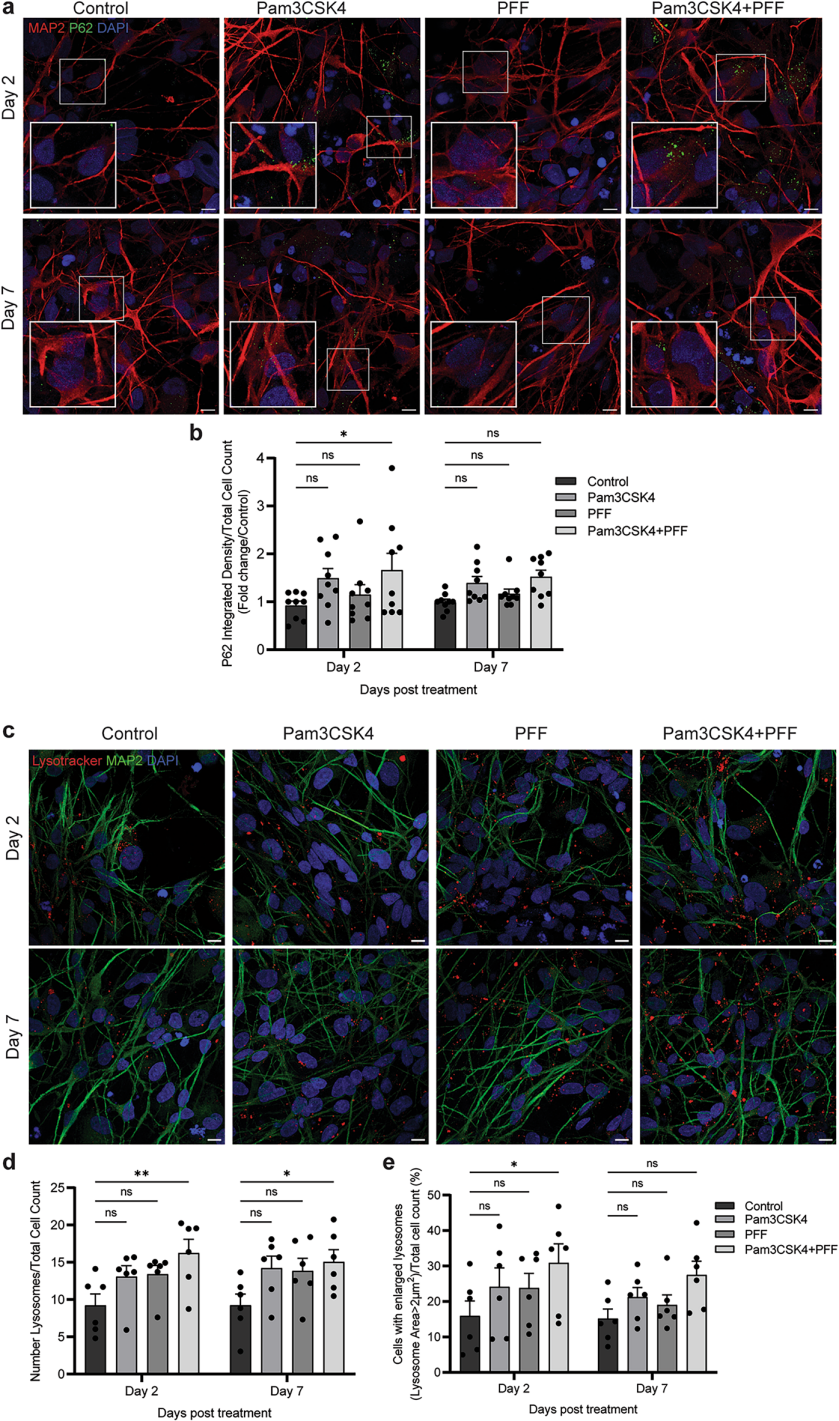
TLR2 activation and treatment with α-syn PFF inhibit autophagy in PD midbrain cells. PD patient iPSCs were differentiated into a midbrain model and cultured for 7 and 14 days with or without 1 μg/ml Pam3CSK4 and/or 1 μg/ml PFFs. **a** Cells were fixed at days indicated and stained for autophagy marker P62 (green) and MAP2 (red). Confocal images were taken at 60 × magnification with 8 images captured per condition and used for the analysis of P62 signal intensity. Scale bar, 10 μm. **b** Graph shows the fold change in P62 intensity/cell as normalised to the control as mean ± SEM (*n* = 6–9). **c** Cells were stained with Lysotracker (red) for 1 h at 37 °C prior to fixation at day 2 and 7 post treatment, and then co-stained for MAP2 (green). Confocal images were taken at 60 × magnification with 8 images captured per condition and used for the analysis. Scale bar, 10 μm. **d, e** Graphs show the number of lysosomes/cell and the percentage of cells with enlarged lysosomes (> 2 μm^2^) respectively, displayed as mean ± SEM (*n* = 6). For all graphs *P < 0.05, **P < 0.01, ns = not significant

As α-syn accumulation following TLR2 activation occurs via inhibition of autophagy in neurons [8, 9], we aimed to assess autophagy inhibition with TLR2 activation in the midbrain model. Differentiated midbrain cells were treated with 1 μg/ml Pam3CSK4 and/or 1 μg/ml α-syn PFFs, fixed on day 2 and day 7 of treatment, and the levels of the autophagy marker P62 measured. When analysing all cell types, P62 was significantly increased on day 2 of treatment with Pam3CSK4 alone but not PFFs (Fig. S3a, b). Consistent with previous observations, following the dual treatment with Pam3CSK4 plus PFF, the levels of P62 were elevated above either treatment alone (Fig. 5a, b), with the levels of P62 declining again by day 7 of treatment. Cells were also stained with Lysotracker and the number and size of lysosomes measured. On days 2 and 7 the number of lysosomes was significantly increased with the Pam3CSK4 plus PFF treatment (Fig. 5c, d), and the percentage of cells with enlarged lysosomes (> 2 μm^2^) was also significantly increased on day 2. On day 7, consistent with levels of P62, the lysosomal size was no longer significantly increased (Fig. 5e). These results are all consistent with a temporary inhibition of autophagy.

### TLR2 activation inhibits autophagy in DA neurons

**Fig. 6:**
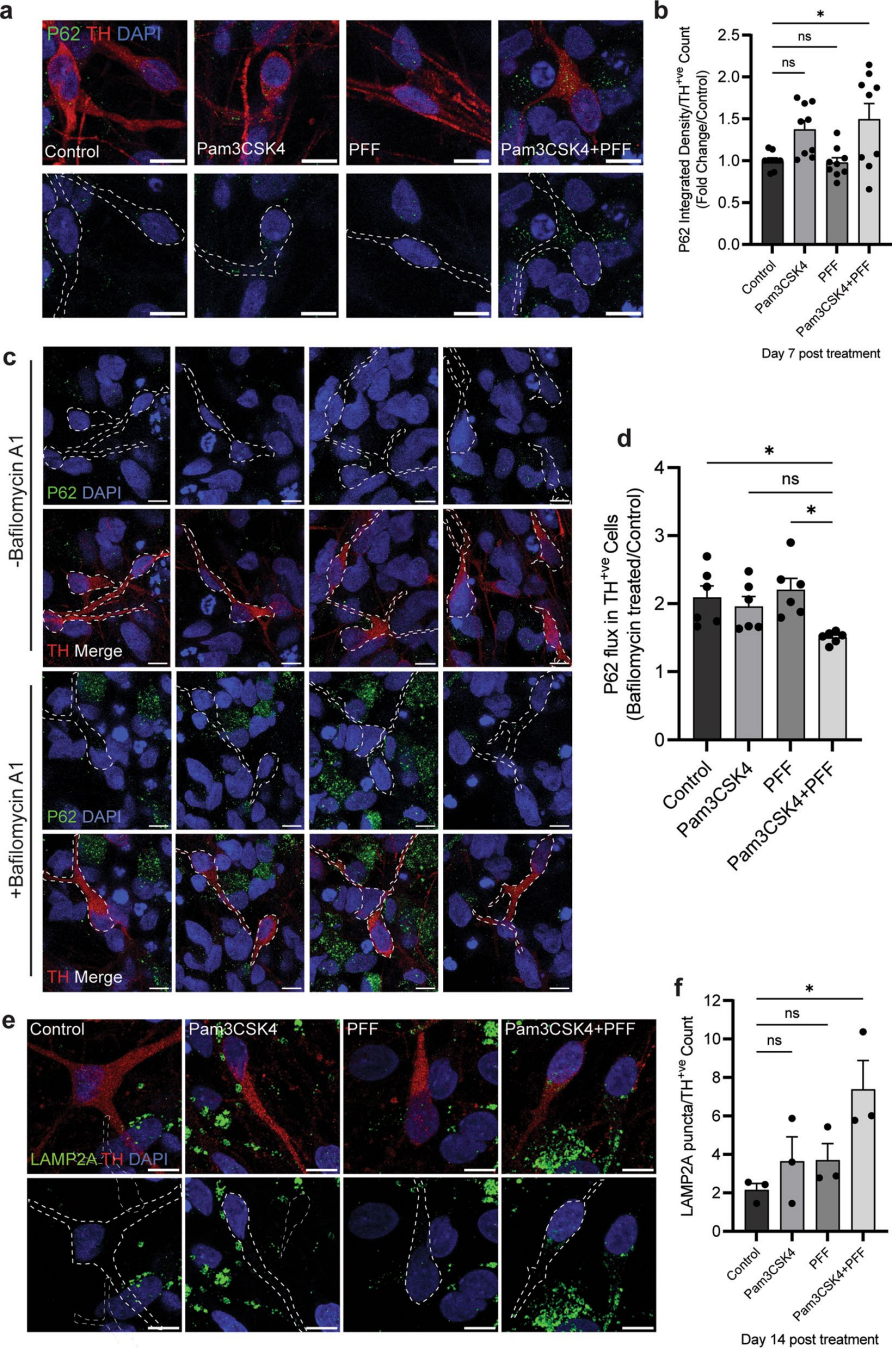
TLR2 activation and α-syn PFF treatment inhibit autophagy in DA neurons. Differentiated midbrain cells derived from PD patient stem cells were treated with 1 μg/ml of Pam3CSK4 and/or 1 μg/ml α-syn PFFs, or medium only for the untreated control, for 7 and 14 days. **a** Cells were fixed at day 7 after treatment and stained for autophagy marker P62 (green) and DA neuron marker TH (red). Confocal images were taken at 60 × magnification with 8 images captured per condition and used for the analysis of P62 signal intensity. Scale bar, 10 μm. **b** Graph shows the fold change in P62 intensity/TH neurons as normalised to the control as mean ± SEM (*n *= 9). **c** To assess autophagy flux cells were treated with Bafilomycin A1 for 4 h, fixed and stained for P62 (green) and TH (red). Confocal images taken at 60 × magnification with 8 images captured per condition. Scale bar, 10 μm. **d** Graph shows autophagy flux measured as P62 expression in Bafilomycin A1 treated/Control treated TH positive neurons, displayed as mean ± SEM (*n* = 6). **e** Cells were fixed at day 14 after treatment and stained for LAMP2A (green) and TH (red). Confocal images were taken at 60 × magnification with 8 images captured per condition. Only signal in TH positive neurons was measured for final analysis. Scale bar, 10 μm. **f** Graph shows the LAMP2A integrated density measured in TH neurons specifically displayed as mean ± SEM (*n* = 3). *P < 0.05, ns = not significant

In contrast to all midbrain cells, when assessing only DA neurons, there was no significant increase in P62 on day 2 of any treatment (Fig. S3c, d). However, on day 7 P62 was significantly increased in DA neurons with the Pam3CSK4 plus PFF treatment (Fig. 6a, b). TLR2 activation alone was sufficient to increase P62 in DA neurons at day 7, but PFFs were not (Fig. S3e, f), and the P62 increase was greater with Pam3CSK4 plus PFFs treatment than with Pam3CSK4 alone. On day 7, autophagy flux was measured in DA neurons following treatment with Bafilomycin A1. Autophagy induction was significantly impaired in the Pam3CSK4 plus PFFs treated cells as observed by decreased levels of P62 (Fig. 6c, d). The CMA marker LAMP2A was also measured in DA neurons at day 14 post-treatment, and was significantly increased with the dual stimulation compared to the untreated cells (Fig. 6e, f).

### Activation of TLR2 leads to autophagy impairment in astrocytes

**Fig. 7:**
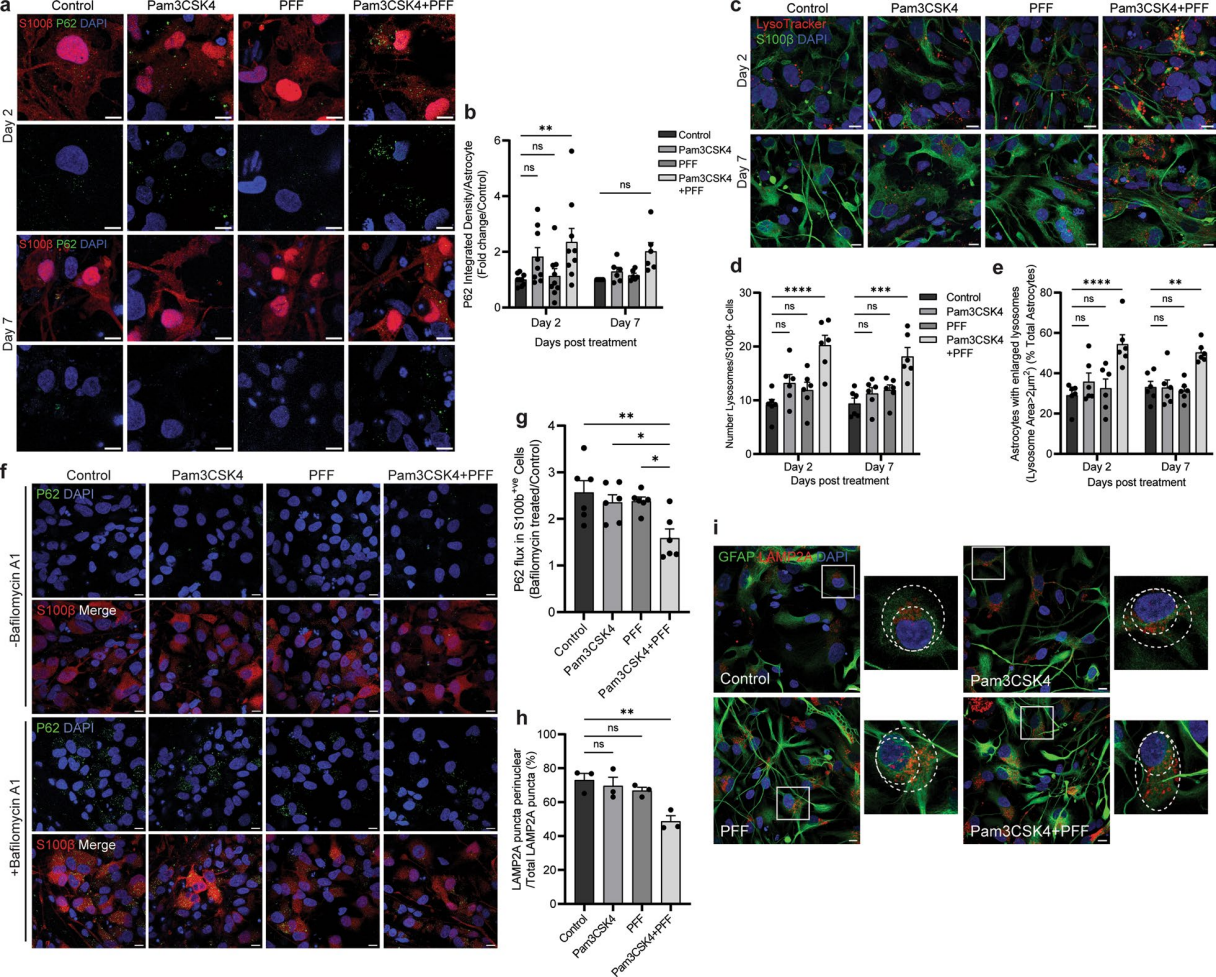
TLR2 activation with α-syn PFF addition inhibits autophagy in astrocytes. Differentiated midbrain cells derived from PD patient iPSCs were treated with 1 μg/ml Pam3CSK4 and/or 1 μg/ml PFFs, or medium only for the untreated control. **a** Cells were fixed at day 2 and 7 after treatment and stained for autophagy marker P62 (green) in S100β positive astrocytes (red). Confocal images were taken at 60 × magnification with 8 images captured per condition, scale bar, 10 μm. **b** Graph shows the fold change in P62 intensity in astrocytes as normalised to the control as mean ± SEM (*n* = 6–9). **c** Cells were stained with Lysotracker (red) for 1 h at 37 °C prior to fixation at day 2 and 7 post treatment, and then co-stained for S100β (green). Confocal images were taken at 60 × magnification with 8 images captured per condition and used for the analysis. Scale bar, 10 μm. **d** Graph shows the average number of lysosomes in astrocytes ± SEM (*n* = 6). **e** Graph shows the percentage of astrocytes with enlarged lysosomes (> 2 μm^2^), displayed by mean ± SEM (*n* = 6).** f** To assess autophagy flux cells were treated with Bafilomycin A1 for 4 h, fixed and stained for P62 (green) and S100β (red). Confocal images were taken at 60 × magnification with 8 images captured per condition. Scale bar, 10 μm. **g** Graph shows autophagy flux measured as P62 expression in Bafilomycin A1 treated/Control treated S100β positive astrocytes, displayed as mean ± SEM (*n* = 6). **h** Graph shows the proportion of LAMP2A puncta in perinuclear positioning as percentage of total LAMP2A puncta measured in astrocytes, displayed mean ± SEM (*n* = 3). **i** Cells were fixed at day 14 after treatment and stained for LAMP2A (red) in GFAP positive astrocytes (green). Confocal images were taken at 60 × magnification with 8 images captured per condition, scale bar, 10 μm. For all graphs **P < 0.01, ***P < 0.001, ****P < 0.0001, ns = not significant

We next aimed to investigate autophagy measures in the astrocyte population in the midbrain model. On day 2, TLR2 activation with Pam3CSK4 treatment alone was sufficient to increase P62 in astrocytes (Fig. S4a, b), but the combination treatment of Pam3CSK4 plus PFFs increased P62 above the untreated control or the Pam3CSK4 treatment alone level (Fig. 7a, b). Similar as seen in all cells, on day 7 the level of P62 in astrocytes was no longer increased when compared to control (Fig. 7a, b). Significant increases of lysosome number and proportion of astrocytes with enlarged lysosomes (> 2 μm^2^) were also detected on day 2 and day 7 after dual treatment (Fig. 7c–e), which were not seen with Pam3CSK4 or PFF treatment alone (Fig. S4c, d). To determine changes in autophagy flux, midbrain cells were treated with Bafilomycin A1 on day 7 of the time course and P62 was measured in astrocytes. Levels of P62 accumulation were significantly decreased in the Pam3CSK4 plus PFF group compared to the control treated astrocytes indicating an impaired autophagy response (Fig. 7f, g). Collectively, these results suggest that the dual treatment leads to not only α-syn aggregation, but also further autophagy impairment in astrocytes. In addition, LAMP2A was measured in this population at day 14 and while no increase in the puncta of LAMP2A was observed with the dual treatment, a significant decrease in the proportion of LAMP2A puncta positioned perinuclearly was seen in astrocytes (Fig. 7h, i), indicating decreased CMA activation [17, 34].

### Co-treatment with Pam3CSK4 and α-syn PFFs increases A1-like astrocytes

**Fig. 8:**
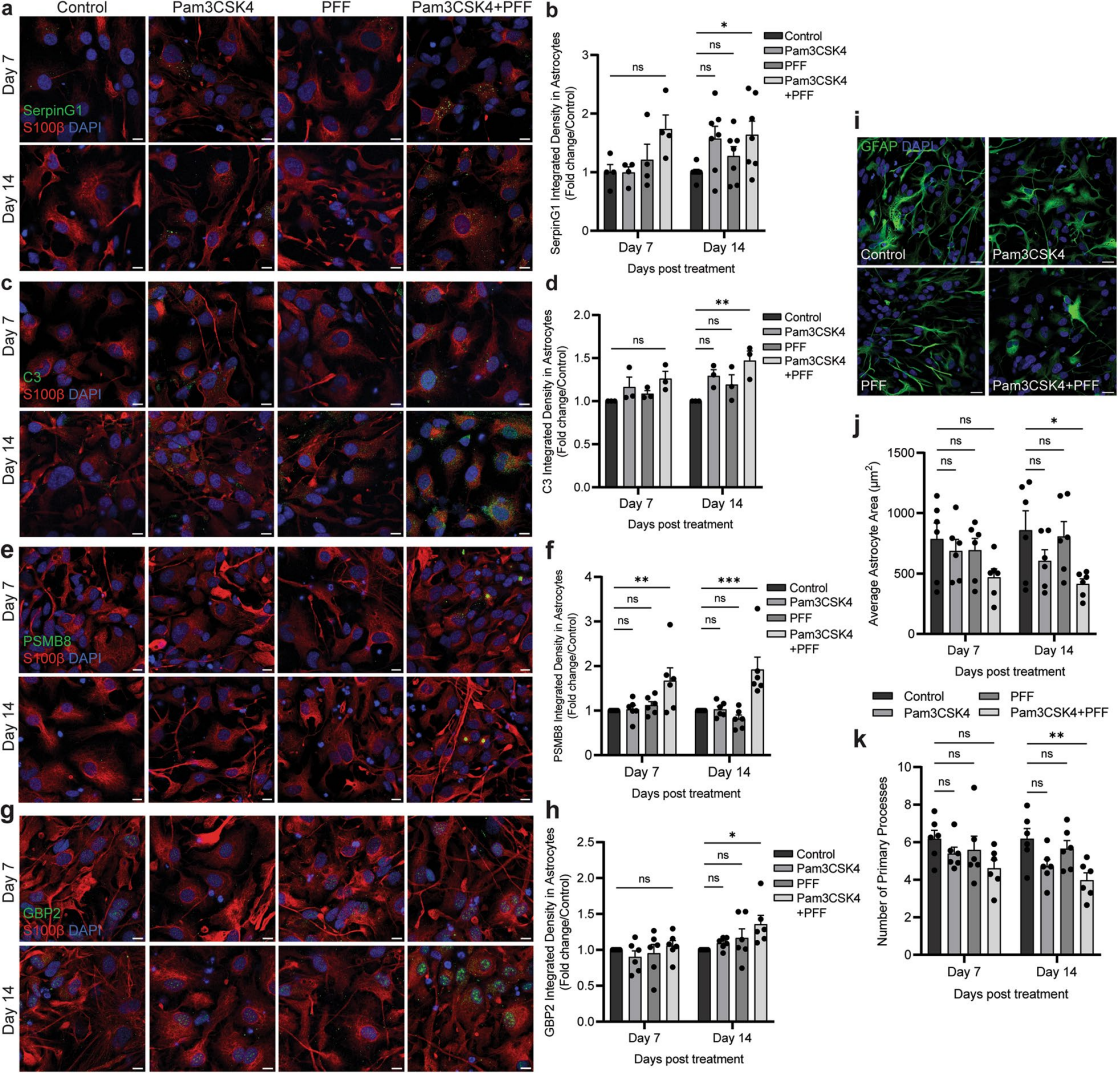
Co-treatment with Pam3CSK4 plus α-syn PFF increases A1 astrocytes. iPSCs derived from PD patients were differentiated into a midbrain model and treated with 1 μg/ml Pam3CSK4 and/or 1 μg/ml PFFs, or medium only for the untreated control for 7 and 14 days. **a**–**h** Cells were fixed at the indicated timepoints after treatment and stained for A1 markers: complement cascade regulator SerpinG1, complement component 3 (C3), PSMB8, or GBP2 (*all in green*) and astrocyte marker S100β (*red*). Confocal images were taken at 40 × magnification with 6–8 images captured per condition and used for signal analysis in astrocytes. Scale bar, 10 μm. Graphs show the expression of A1 markers measured in S100β positive astrocytes expressed as fold change/control, displayed as mean ± SEM (*n* = 3–7). **i** cells were fixed at the indicated timepoints after treatment and stained for astrocyte marker GFAP (*green*). Confocal images were taken at 40 × magnification with 6 images captured per condition. Scale bar, 20 μm. **j** graph shows average astrocyte area (μm^2^) as mean ± SEM (*n* = 6). **k** graph shows number of primary processes extending from astrocyte soma displayed as mean ± SEM (*n* = 6). For all graphs *P < 0.05, **P < 0.01, ***P < 0.001, ns = not significant

Astrocytes in PD show changes associated with an A1 phenotype, including upregulation of complement cascade proteins such as complement component 3 (C3), and more broadly upregulation of SerpinG1, and morphological changes such as decreased area [19]. We therefore investigated if treatment with Pam3CSK4 plus PFF was associated with the astrocytic A1 phenotype. Astrocytes displayed a significant increase in the expression of SerpinG1 (Fig. 8a, b), complement C3 (Fig. 8c, d), PSMB8 (Fig. 8e, f), and GBP2 (Fig. 8g, h) on day 14 after the treatment with Pam3CSK4 plus PFF when compared to the control. Additionally, on day 14, the astrocyte area and the number of primary processes from the soma were significantly decreased (Fig. 8i–k). Notably, the decreases of astrocyte area and primary processes as well as increased PSMB8 expression were seen on day 7, indicating that the changes associated with the A1-like phenotype are commencing at this earlier time point. In addition, astrocyte activation markers S100β and GFAP were measured, with no differences observed between the treatment conditions (Fig. S5a–c).

### Treatment with Pam3CSK4 plus PFF leads to DA neuronal loss in a midbrain model

**Fig. 9:**
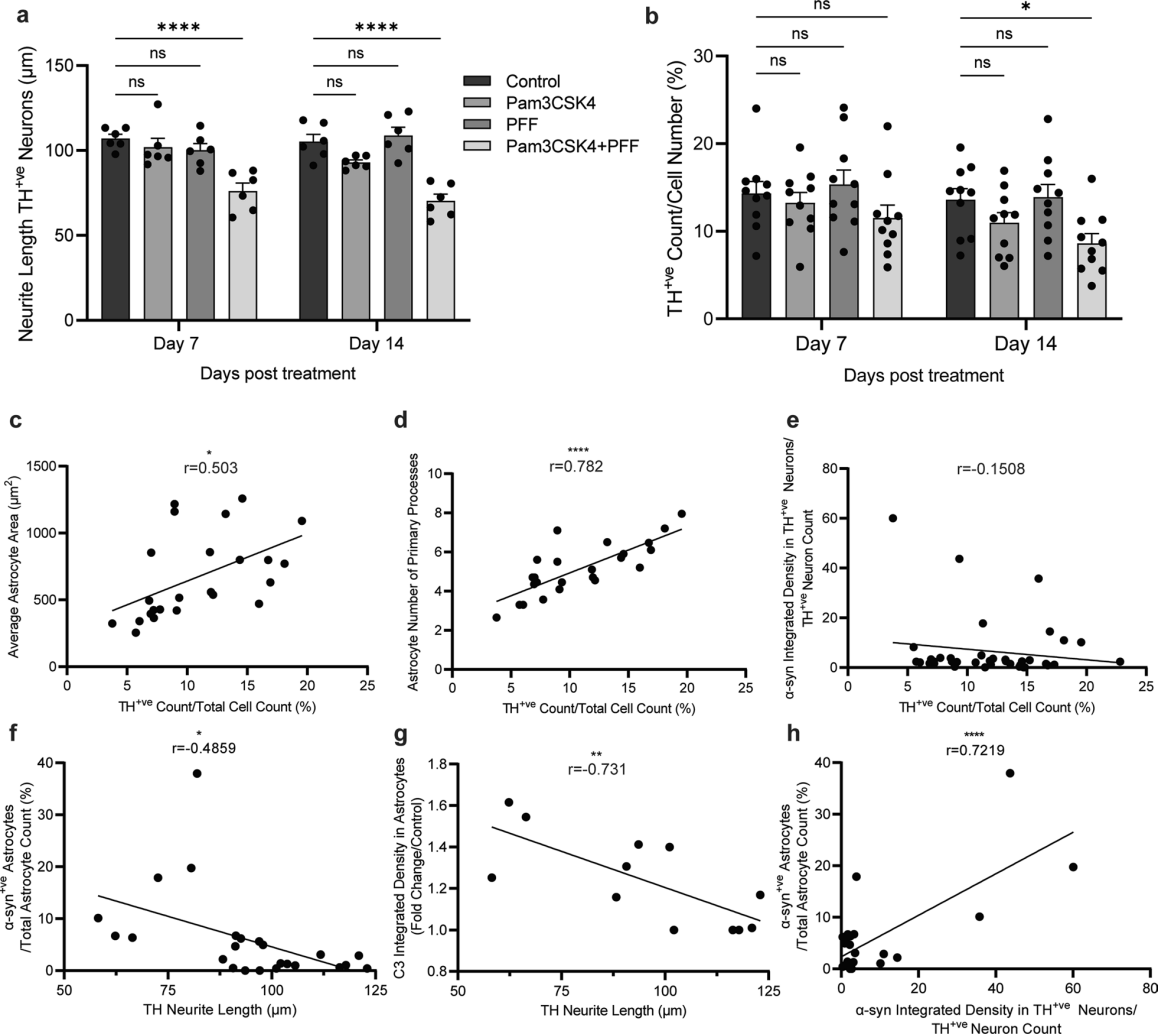
Specific loss of DA neurons with Pam3CSK4 plus α-syn PFF treatment. Differentiated midbrain cells derived from PD patient iPSCs were cultured for 7 and 14 days with 1 μg/ml Pam3CSK4 and/or 1 μg/ml PFFs, or medium only for the untreated control. **a** Graph shows the length of neurites extending from the soma of TH-positive neurons (μm) as mean ± SEM. 30 cells in each replicate were included in the analysis (*n* = 6). **b** Graph shows the number of TH positive neurons/DAPI (%) displayed as mean ± SEM (*n* = 10). **c**–**h** Graphs show Pearson correlation between two variables, as described on the axis. For all graphs *P < 0.05, **P < 0.01, ****P < 0.0001, ns = not significant

As astrocyte function is important for maintaining DA neuronal health, we next aimed to assess neuronal health in the midbrain model. DA neurons displayed a significant decrease in neurite length on day 7 and day 14 of combination treatment with Pam3CSK4 plus PFF (Fig. 9a with tracing analysis shown in Fig. S6), suggesting degeneration of this population. Indeed, there was a significant decrease in the number of DA neurons on day 14 following the treatment with Pam3CSK4 plus PFF (Fig. 9b). Notably, the levels of LDH released in the medium did not change in the Pam3CSK4 plus PFF treated cells compared to the control, Pam3CSK4 or PFF treated group (Fig. S7a), suggesting that cell death was not widespread. In addition, the number of S100β-positive cells and the area of MAP2 were not altered in any of the treatment conditions (Fig. S7b–d).

To identify possible contributing factors to the loss of DA neurons in this model, correlations of the measures of DA neuronal health, cell number and neurite length with A1 astrocyte phenotype and α-syn pathology measures were analysed. The number of DA neurons significantly correlated positively with the astrocyte area and the number of primary processes (Fig. 9c, d). However, the number of DA neurons had a weak correlation with α-syn signal in DA neurons (Fig. 9e). The neurite length of DA neurons showed significant negative correlations with the percentage of astrocytes with α-syn aggregates and the complement C3 signal measured in astrocytes (Fig. 9f, g). Additionally, α-syn measured in DA neurons displayed a significant positive correlation with the percentage of astrocytes with α-syn aggregates (Fig. 9h). Overall, the measures of astrocyte A1 phenotype and astrocytic α-syn accumulation in this model strongly correlated with DA neuronal loss, indicating that astrocyte dysfunction contributes to DA neuronal loss.

## Discussion

A midbrain model incorporating astrocytes, neurons and DA neurons was generated from three control and three PD patient stem cell lines using a published protocol [28] and characterised in the present study. Using this model, we were able to investigate the development of α-syn pathology mediated by TLR2 activation and the effect on autophagy, DA neuronal health and astrocyte activation. TLR2 activation resulted in the inhibition of autophagy and propagation of α-syn pathology in neurons and astrocytes. Astrocytes also displayed morphological characteristics and upregulation of proteins consistent with a shift to an A1 pro-inflammatory phenotype. The shift of astrocytes to the A1 phenotype was associated with a reduction of DA neurons specifically.

In the midbrain model presented, 45%–50% of cells were S100β-positive astrocytes and 50%–55% were MAP2-positive neurons, consistent with previous reports of the midbrain culture composition [28, 35]. In addition, 10%–20% of cells were TH-positive, which aligns with the expected percentage observed in previously published work [17, 36, 37], but the percentage differed significantly between PD lines, which has been reported due to differences in the differentiation potential of neural progenitor cells derived from PD patients [38–41]. In human brains, transcriptome profiling of the SN has reported that astrocytes represent about 12% of all glia (80% of all cells), and DA neurons account for a small population of only about 1.21% of all cells [42], with a similar proportion of cells also reported in the human midbrain [43]. Stem cell-derived midbrain models are decreased in the complexity of cellular composition and therefore the ratio of astrocytes to DA neurons is increased when compared to the human brain. Nonetheless, these models enable investigation into the contribution of astrocyte dysfunction to DA neuron health.

Treatment of the midbrain cells with a combination of a TLR2 agonist and α-syn fibrils significantly increased α-syn pathology and decreased the neurite length in DA neurons on day 7, but loss of this population was only observed at the later time point of day 14. TLR2 activation alone was sufficient to increase P62 in DA neurons at day 7, suggesting autophagy inhibition, but the increase in α-syn and shortening of neurites were only seen with the combination of TLR2 activation plus α-syn fibrils, suggesting that the inhibition of autophagy in DA neurons is not sufficient to induce neurodegeneration. Previous reports have shown that the accumulation of α-syn can lead to the selective death of DA neurons [44, 45]. LB pathology is seen in surviving neurons in PD, but the formation of insoluble aggregates is not always observed when DA neurons are lost [44], indicating that the formation of α-syn pathology is not the only contributing factor to the loss of this neuronal population. Indeed, recent reports have highlighted a role for astrocytic dysfunction in the loss of DA neurons. The co-culture of iPSC-derived mutant PD astrocytes and control DA neurons leads to a decrease of DA neurons and increased α-syn in surviving DA neurons due to altered autophagy and α-syn accumulation in PD astrocytes [17]. A more recent study observed that the treatment of iPSC-derived DA neurons with conditioned culture medium from iPSC-derived PD astrocytes carrying a *LRRK2* mutation induced DA neuronal death through increased α-syn in multivesicular bodies prior to their secretion, and decreased secretion of neurotrophic factors from astrocytes [46]. In agreement with previous work, neuronal loss was limited to DA neurons and the population of MAP2^+^/TH^−^ neurons was not altered with the increase of α-syn aggregation and astrocyte dysfunction. However, the selective vulnerability of DA neurons and whether α-syn aggregation alone can lead to neuronal death remain unclear. However, a reduction in the neuronal supportive functions of astrocytes is hypothesised to contribute to the degeneration of DA neurons.

The data presented in this study support a role for astrocyte dysfunction in the loss of DA neurons, in the inhibition of autophagy and in α-syn accumulation. Given the low expression of α-syn in astrocytes, it can be hypothesised that the accumulation of α-syn seen in the co-treatment with TLR2 activation and PFFs results from reduced capacity of astrocytes to degrade what is endocytosed from exogenous α-syn PFFs or neuronally-derived α-syn, leading to increased α-syn aggregation. Indeed, astrocytes displayed increases of lysosome number and number of astrocytes with enlarged lysosomes, as well as a decreased proportion of perinuclear LAMP2A, indicating decreased autophagy and CMA specifically. Astrocytes are normally able to degrade fibrillar α-syn, and α-syn transferred from astrocytes to astrocytes or neurons to astrocytes [47], suggesting an active role for astrocytes in the clearance of α-syn. Degradation of α-syn is done largely by CMA mediated by LAMP2A, but larger aggregates have been reported to be unable to be degraded via this pathway [48–50], and accumulation of excess α-syn in astrocytes can disrupt the lysosomal pathway [51]. In addition, previous reports suggest that TLR2 stimulation in astrocytes results in higher uptake of α-syn fibrils, and when pre-treated with α-syn fibrils, TLR2 activation delays degradation in astrocytes, leading to increased α-syn accumulation [52]. Astrocytes displayed higher expression of TLR2 compared to neurons, and Pam3CSK4 treatment further upregulated TLR2 in this population, which lead to autophagy inhibition in astrocytes. This likely decreased the degradation of α-syn in astrocytes, enabling its accumulation, which further exacerbated the disruption of lysosomal pathways, leading to astrocytic dysfunction and a loss of supportive functions for DA neurons.

The accumulation of α-syn and lysosomal impairment in astrocytes in the co-treatment with TLR2 activation and PFFs may also contribute to the A1-associated changes of astrocytes. It has mainly been reported that the shift to A1 astrocytes is driven by the pro-inflammatory response of microglia [19, 22]. However, in the absence of microglia, Liddelow and colleagues demonstrated that the treatment of astrocytes in culture with pro-inflammatory cytokines TNF-α, IL-1α and C1q is capable of inducing an A1 phenotype similar to that induced by treatment with conditioned medium from activated microglia, and treatment with only one cytokine can upregulate some A1-associated genes such as *SerpinG1* [19]. In addition, direct exposure to α-syn PFFs in vitro upregulated expression of SerpinG1 and PSMB8 in astrocytes [23], and treatment with α-syn PFFs in vivo increased complement C3 expression [22]. Aggregated α-syn can act as a TLR2 ligand, inducing the expression of TNF-α and IL-1β [53, 54], and exposure of astrocytes to neuronally derived α-syn in vitro increased the expression of inflammatory genes, such as TNF-α, IL-1α and IL-1β [18]. Notably, A1 astrocytes are observed in PD as well as in cases of normal aging [19, 55], suggesting that reduced astrocytic functions in the promotion of neuronal survival may contribute to neuron degeneration and neuroinflammation in disease. Moreover, the pro-inflammatory pathway(s) activated by TLR2 activation and α-syn aggregation in this study may induce a partial shift to an A1-like phenotype as seen with upregulation of A1-associated proteins in astrocytes as well as morphological changes, although these changes were not seen until later in the time course, indicating this shift occurs only with prolonged exposure to α-syn aggregates or a chronic inflammatory response. However, the role of astrocytes in the accumulation and propagation of α-syn in PD remains unclear. Astrocytes may act to phagocytose α-syn to remove neuronal waste or directly contribute to the spread of neuronal α-syn via uptake and propagation of the phagocytosed material, increasing neuronal α-syn [56, 57]. The capacity of astrocytes to clear accumulated α-syn and their activation status possibly determine the role of astrocytes in the progression of PD, whether it is beneficial in the clearance of α-syn or harmful in contributing to the spread of α-syn pathology. Moreover, the question remains as to the contribution of A1 astrocytes to the specific loss of DA neurons, a possibility in the context of sustained loss of normal functions such as degradation of neuronal waste and secretion of neurotropic factors. Further work is needed to investigate the progressive shift of astrocytes to the A1 phenotype in PD, the stimuli driving this shift, and the selective vulnerability of DA neurons.

Overall, the findings of this study support the hypothesis of a role for astrocytic dysfunction in the loss of DA neurons in PD. In this midbrain model, α-syn pathology was observed not only in neurons but also in astrocytes, with alterations in autophagy pathways as well as a pro-inflammatory shift of astrocyte phenotypes that contributed to DA neuronal decline. Dysfunctional astrocytes may mediate DA neuronal loss through the loss of supportive functions. Future work using orthogonal approaches to inhibit the autophagy-lysosome pathway is required to determine the signalling mechanism(s) and the contribution of autophagy inhibition with TLR2 activation in astrocytes. This study also used the synthetic TLR2 agonist Pam3CSK4, while the exact physiological trigger for TLR2 activation in PD brains remains to be determined. It would also be advantageous to confirm TLR2-dependent effects in the midbrain model by knocking out *TLR2* and/or using TLR2 antagonists as we have previously done using SH-SY5Y neuroblastoma cells [9]. Future studies should also aim to elucidate the temporal shift to A1 astrocytes in the PD brain, and how this may contribute to neuroinflammation and DA neuronal death, including the addition of other inflammatory markers. As microglia also respond to TLR2 agonists, further development of the midbrain model to include microglia would also be important for determining how TLR2 activation influences the internalization of fibrils in different cell types.

## Conclusions

TLR2 is emerging as a potential therapeutic target for the treatment of PD. This work demonstrates that activation of TLR2 inhibits autophagy and exacerbates α-syn pathology in both neurons and astrocytes. This results in pro-inflammatory activation of astrocytes, which is associated with specific loss of DA neurons in a human cellular midbrain model. These findings support work in cell and animal models indicating that TLR2 inhibition may be beneficial for the treatment of PD.

## Supplementary Information

**Additional file 1.** **Figure S1**. Midbrain cells were lysed for immunoblot detection of neuronal specific proteins MAP2 and β-III-tubulin, astrocyte marker S100β, and proteins α-syn, P62, and TLR2.** Figure S2**. Representative plots of flow cytometry gating strategy to measure TLR2 in the different cell populations in the midbrain model. **Figure S3**. Treatment of differentiated midbrain cells with 1 μg/mL of Pam3CSK4 only increased autophagy marker P62 levels compared to the control, and α-syn PFFs did not. **Figure S4.** TLR2 activation with Pam3CSK4 treatment alone increased P62 in astrocytes compared to the untreated control, and α-syn PFFs did not.** Figure S5**. Treatment of Pam3CSK4 and α-syn PFFs did not increase expression of classical astrocyte activation markers. **Figure S6**. Midbrain cells were treated with Pam3CSK4 and/or a-syn PFFs, fixed at days 7 and 14 and stained for TH (green) for analysis of neurite length in DA neurons. **Figure S7**. Treatment of Pam3CSK4 plus PFFs did not increase LDH released into the culture media or decrease MAP2 area. **Table S1**. List of antibodies used in this study.

## Data Availability

All data generated or analysed during this study are included in this published article and its supplementary information files.

## Abbreviations

PD: Parkinson’s disease
α-syn: Alpha-synuclein
TLR2: Toll-like receptor 2
LB: Lewy Body
DA: Dopaminergic
PFF: Pre-formed fibril
CMA: Chaperone-mediated autophagy
