# Supplementary material for: Astrocytes contribute to toll-like receptor 2-mediated neurodegeneration and alpha-synuclein pathology in a human midbrain Parkinson’s model
Source: Transl Neurodegener. 2024 Dec 16;13:62. doi: 10.1186/s40035-024-00448-3 (PMC11648304; doi:10.1186/s40035-024-00448-3)
Supplement: Supplementary file 1 — Additional file 1. Figure S1. Midbrain cells were lysed for immunoblot detection of neuronal specific proteins MAP2 and β-III-tubulin, astrocyte marker S100β, and proteins α-syn, P62, and TLR2. Figure S2. Representative plots of flow cytometry gating strategy to measure TLR2 in the different cell populations in the midbrain model. Figure S3. Treatment of differentiated midbrain cells with 1 μg/mL of Pam3CSK4 only increased autophagy marker P62 levels compared to the control, and α-syn PFFs did not. Figure S4. TLR2 activation with Pam3CSK4 treatment alone increased P62 in astrocytes compared to the untreated control, and α-syn PFFs did not. Figure S5. Treatment of Pam3CSK4 and α-syn PFFs did not increase expression of classical astrocyte activation markers. Figure S6. Midbrain cells were treated with Pam3CSK4 and/or a-syn PFFs, fixed at days 7 and 14 and stained for TH (green) for analysis of neurite length in DA neurons. Figure S7. Treatment of Pam3CSK4 plus PFFs did not increase LDH released into the culture media or decrease MAP2 area. Table S1. List of antibodies used in this study. [file 40035_2024_448_MOESM1_ESM.pdf]

Astrocytes contribute to TLR2-mediated neurodegeneration and alpha-synuclein pathology in a human midbrain Parkinson's model

Fiona Weiss[1], Laura Hughes[1], Yuhong Fu[1], Cedric Bardy[2], Glenda M Halliday[1], Nicolas Dzamko[1]*

[1]School of Medical Sciences, Faculty of Medicine and Health and the Brain and Mind Centre, University of Sydney, Camperdown, NSW, 2050, Australia.

[2]South Australian Health and Medical Research Institute (SAHMRI), Laboratory for Human Neurophysiology and Genetics, Adelaide, SA, Australia

***Correspondence**:

Nicolas Dzamko

nicolas.dzamko@sydney.edu.au
Keywords: Parkinson's disease, Toll-like receptor 2, alpha-synuclein, astrocyte

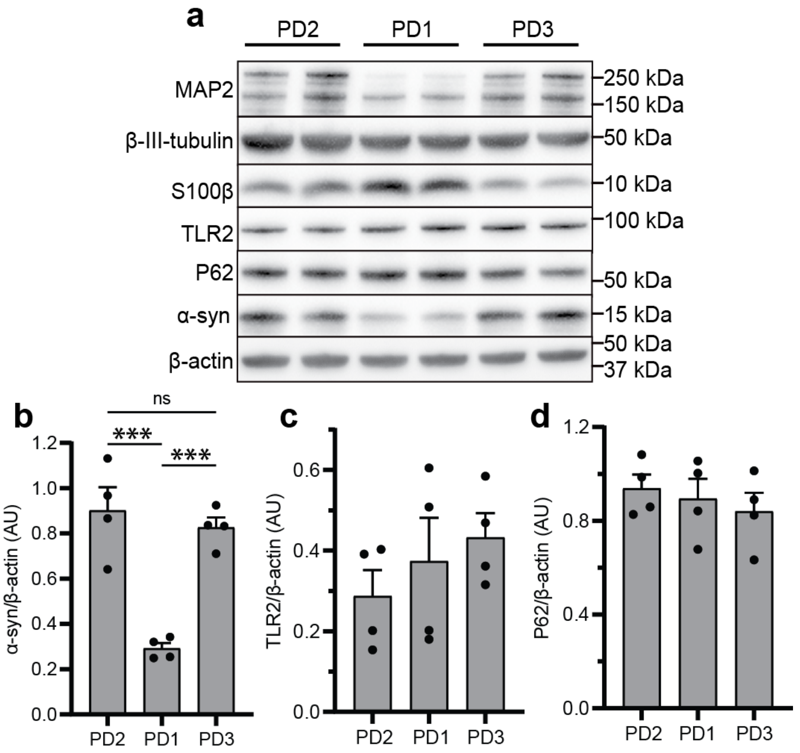

Figure S1: **a**, Midbrain cells were lysed for immunoblot detection of neuronal specific proteins MAP2 and β-III-tubulin, astrocyte marker S100β, and proteins α-syn, P62, and TLR2. β-actin was used as a loading control. **b-d**, graphs show quantification of α-syn, TLR2 and P62 normalised to β-actin respectively, displayed as mean ± S.E.M

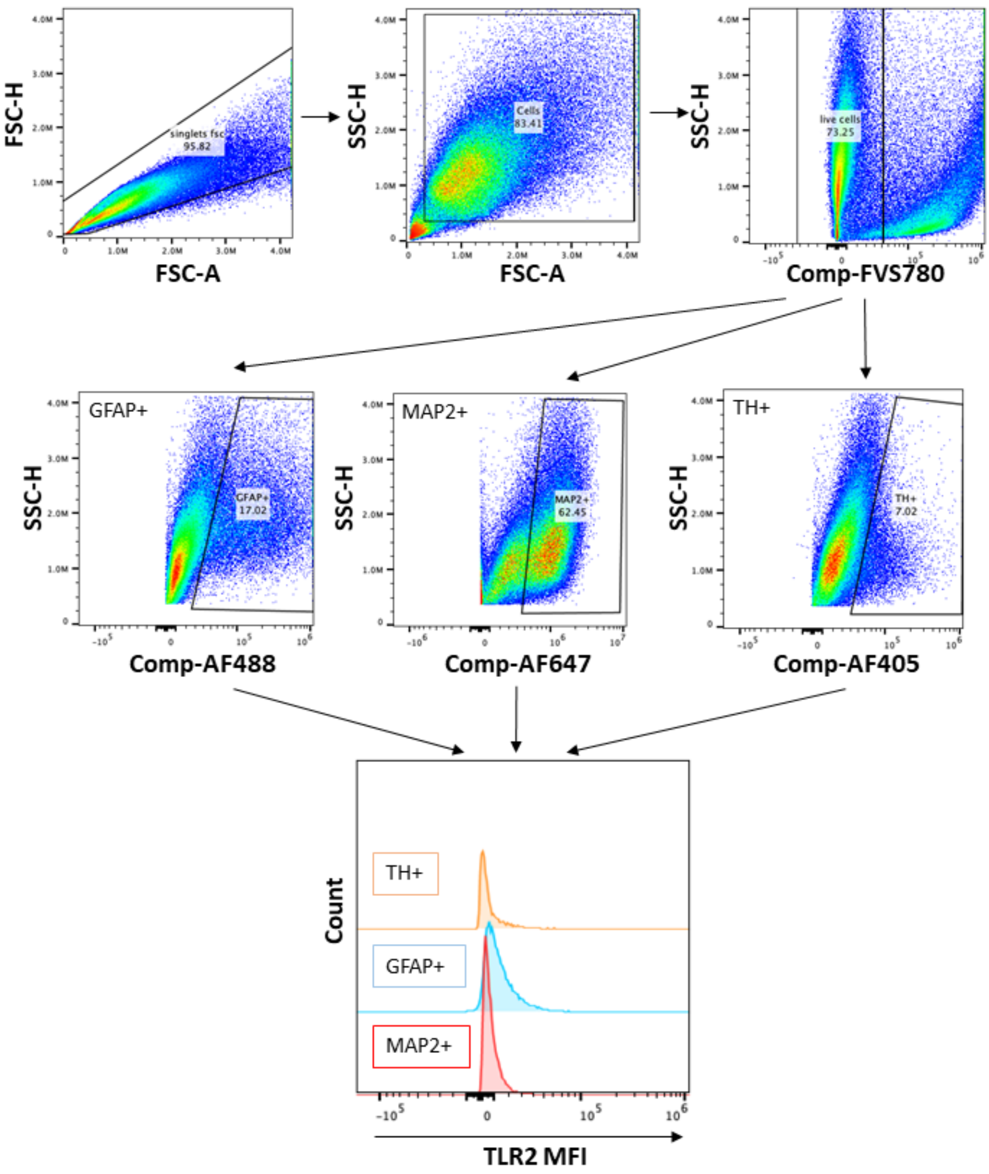

Figure S2: Representative plots of flow cytometry gating strategy to measure TLR2 in the different cell populations in the midbrain model. Differentiated midbrain cells were harvested with Accutase, stained for flow cytometry analysis and samples run on the Cytek Aurora spectral analyser with a minimum of 200 000 events captured per sample (n=3). **a**, firstly a singlet gate was created using forward scatter area (FCS-A) and forward scatter height (FSC-H) parameters to exclude doublets. **b**, this was followed by a cell specific gate based on FSC-A and side scatter height (SSC-H) parameters to omit debris in samples from final analysis. **c**, next a gate was created to capture only live cells based on the live/dead stain (Fixed viability stain 780). **d-f**, Within the live cell category the cells were further categorised into cells positive for GFAP, MAP2 and TH. **g**, in these cell populations the median fluorescence intensity (MFI) of TLR2 was measured.

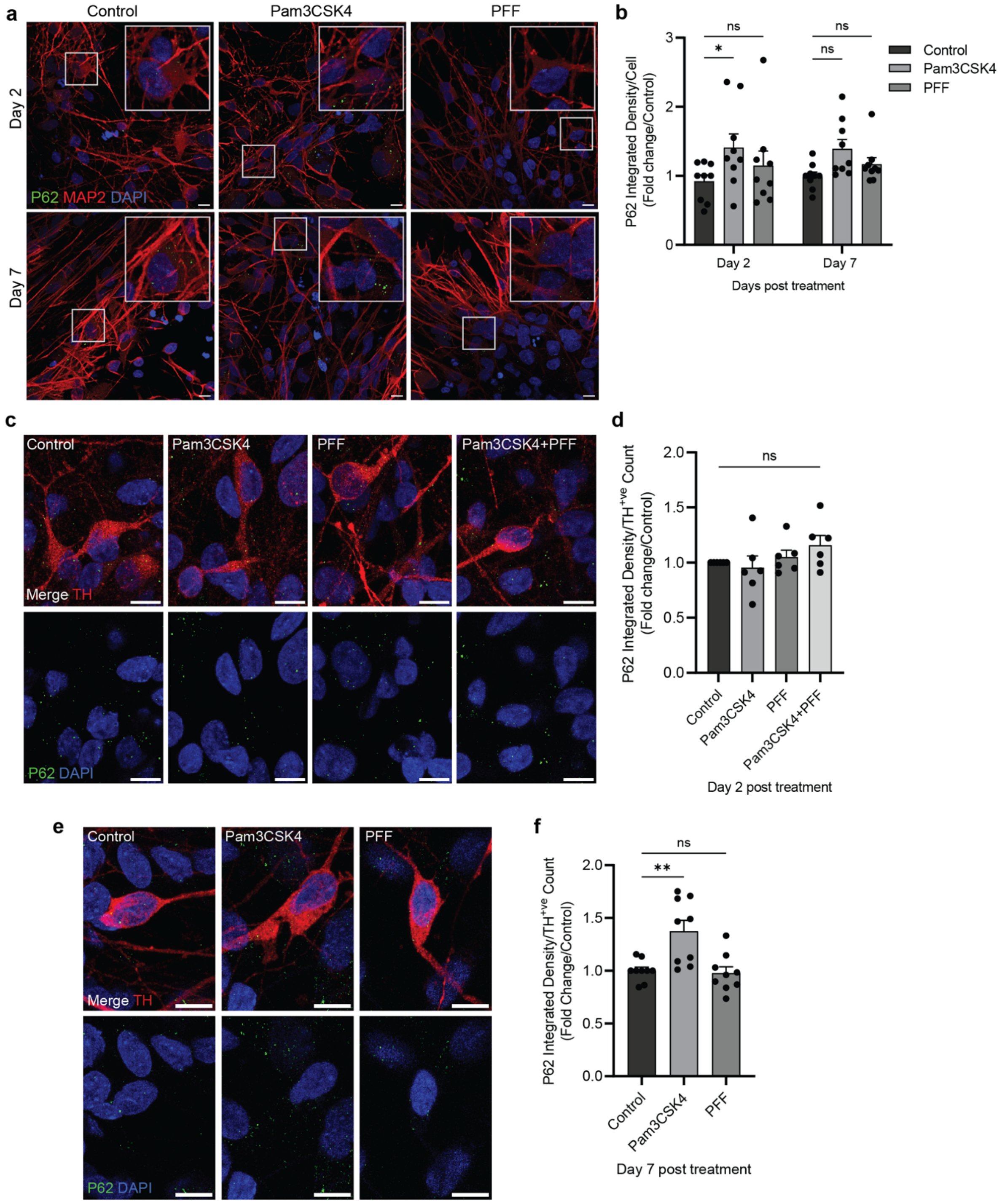

Figure S3: Treatment of differentiated midbrain cells with 1 µg/mL of Pam3CSK4 only increased autophagy marker P62 levels compared to the control, and α-syn PFFs did not. At day 2 no increase in P62 was observed with any treatment in TH positive neurons, but at day 7 Pam3CSK4 treatment alone increased P62 significantly compared to the control, and PFFs did not. Results from figure 4B and 5B were reanalysed without the combination treatment of Pam3CSK4 plus PFFs to show significant increases in P62 with TLR2 activation alone in all cells and DA neurons specifically. **A**, cells were fixed at day 2 and 7 after treatment and stained

for P62 (*green*) and MAP2 (*red*). Confocal images were taken at 60X magnification with 8 images captured per condition and used for the analysis of P62 signal intensity. Scale bar=10 µm. **b**, graph shows the fold change in P62 intensity/cell as normalised to the control, displayed as mean ± S.E.M (n=9). **c**, cells were fixed at day 2 after treatment and stained for P62 (*green*) and TH (*red*). Confocal images were taken at 60X magnification with 8 images captured per condition and used for the analysis of P62 signal intensity. Scale bar=10 µm. **d**, graph shows the fold change in P62 intensity/cell as normalised to the control as mean ± S.E.M (n=6). **e**, cells were fixed at day 7 after treatment and stained for P62 (*green*) and TH (*red*). Confocal images were taken at 60X magnification with 8 images captured per condition and used for the analysis of P62 signal intensity. Scale bar=10 µm. **f**, graph shows the fold change in P62 intensity/cell as normalised to the control as mean ± S.E.M (n=9). For all graphs *p <0.05, ns=not significant.

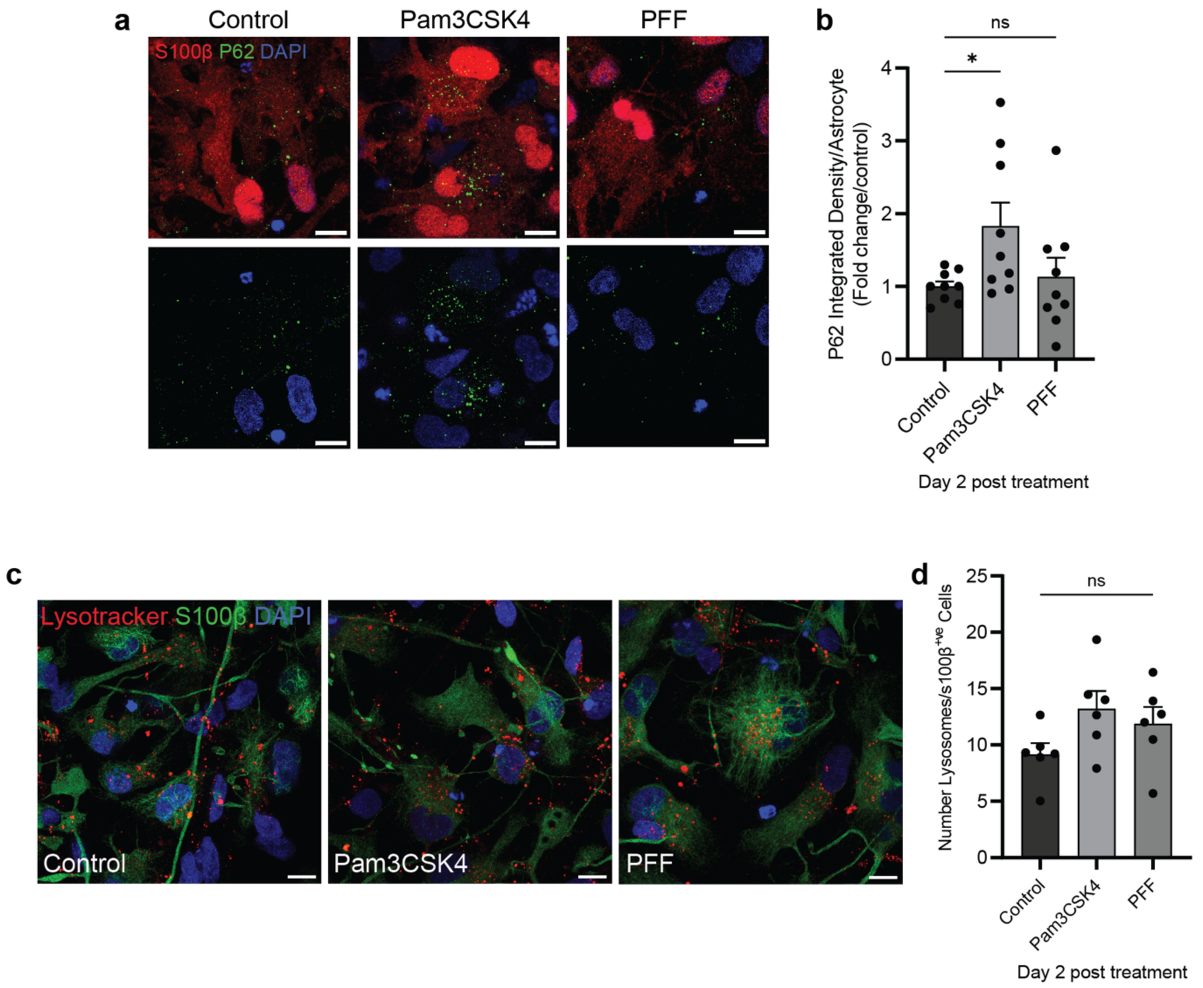

Figure S4: TLR2 activation with Pam3CSK4 treatment alone increased P62 in astrocytes compared to the untreated control, and α-syn PFFs did not. Results from figure 6B and 6D were reanalysed without the combination treatment to show differences with Pam3CSK4 treatment alone. However, the number of lysosomes in astrocytes was not increased with Pam3CSK4 or PFFs alone regardless of the removal of the treatment condition Pam3CSK4 plus PFF. **a**, cells were fixed at day 2 after treatment and stained for P62 (*green*) and S100β (red). Confocal images were taken at 60X magnification with 8 images captured per condition and used for analysis. Scale bar =10 µm. **b**, graph shows P62 signal/astrocyte (S100β positive cell), displayed as mean ± S.E.M (n=9). **c,** cells were stained with Lysotracker (*red*) for 1 h at 37°C prior to fixation at day 2 post treatment, and then co-stained for astrocyte marker S100β (*green*). Confocal images were taken at 60X magnification with 8 images captured per condition and used for the analysis of the number of lysosomes in astrocytes. Scale bar=10 µm. **d**, graph shows analysis of the number of lysosomes in astrocytes as mean ± S.E.M (n=6). *p<0.05, ns=not significant.

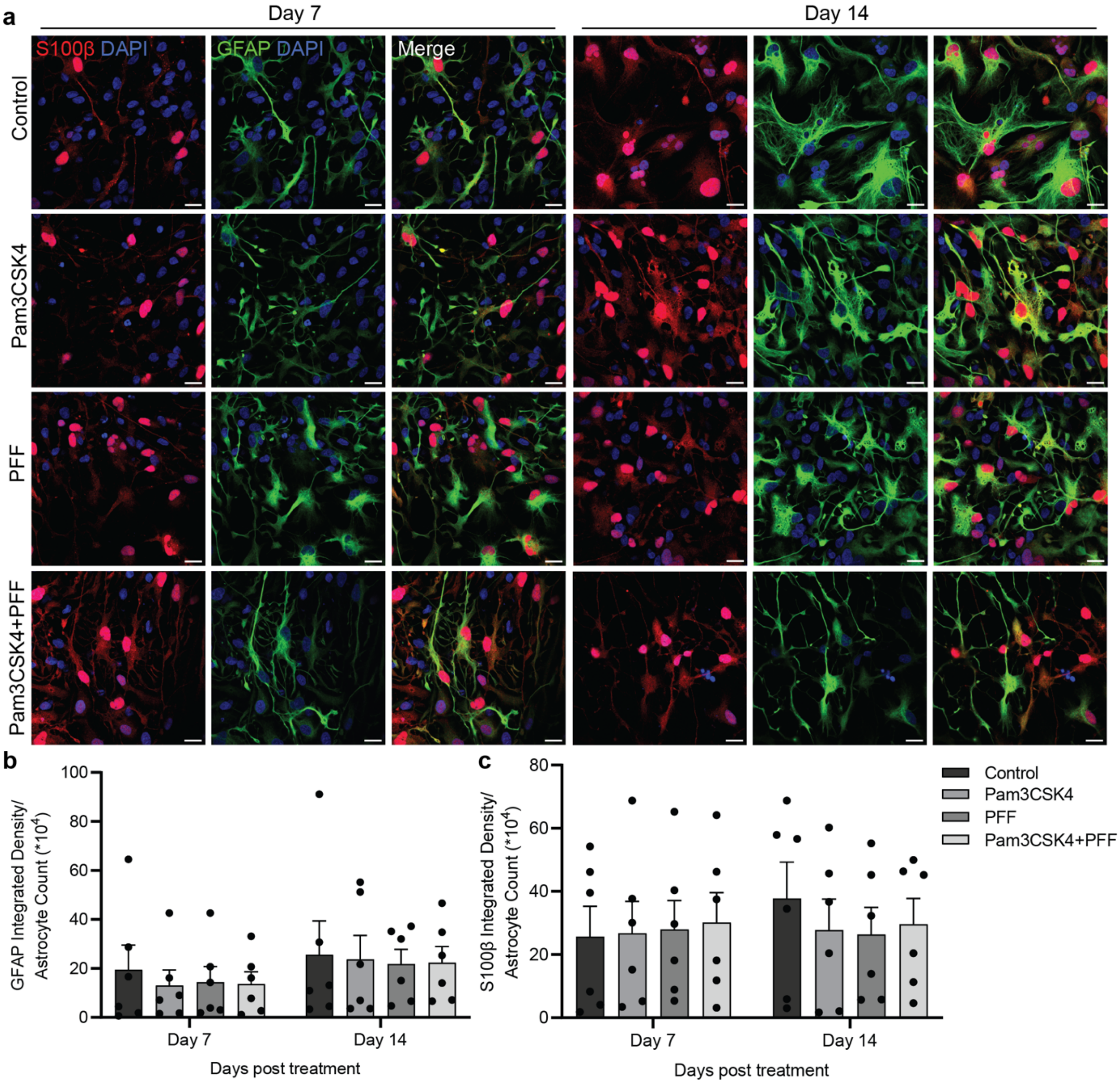

Figure S5: Treatment of Pam3CSK4 and α-syn PFFs did not increase expression of classical astrocyte activation markers. **a**, midbrain cells were treated with Pam3CSK4 and/or α-syn PFFs, or media only for the untreated control, fixed at day 7 and 14 and stained for astrocyte markers S100β (*red*), GFAP (*green*) and DAPI stains the nuclei (*blue*). Confocal images taken at 40X magnification with 6 images taken per condition, scale bar=20 µm. **b-c**, graphs show image analysis of GFAP and S100β expression respectively, displayed as mean ± S.E.M. (n=6).

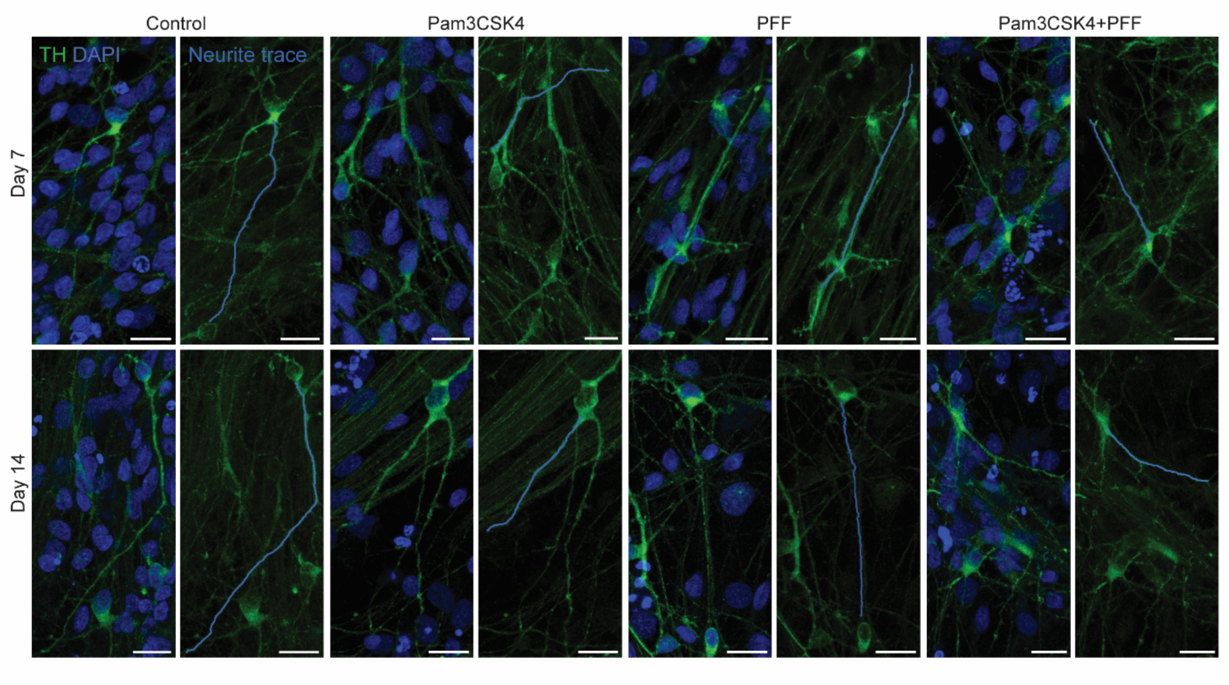

Figure S6: Midbrain cells were treated with Pam3CSK4 and/or a-syn PFFs, fixed at days 7 and 14 and stained for TH (*green*) for analysis of neurite length in DA neurons. Images taken on confocal microscope with 40X magnification, scale bar=20 µm. The SNT plugin in Fiji ImageJ was used for neurite tracing and analysis.

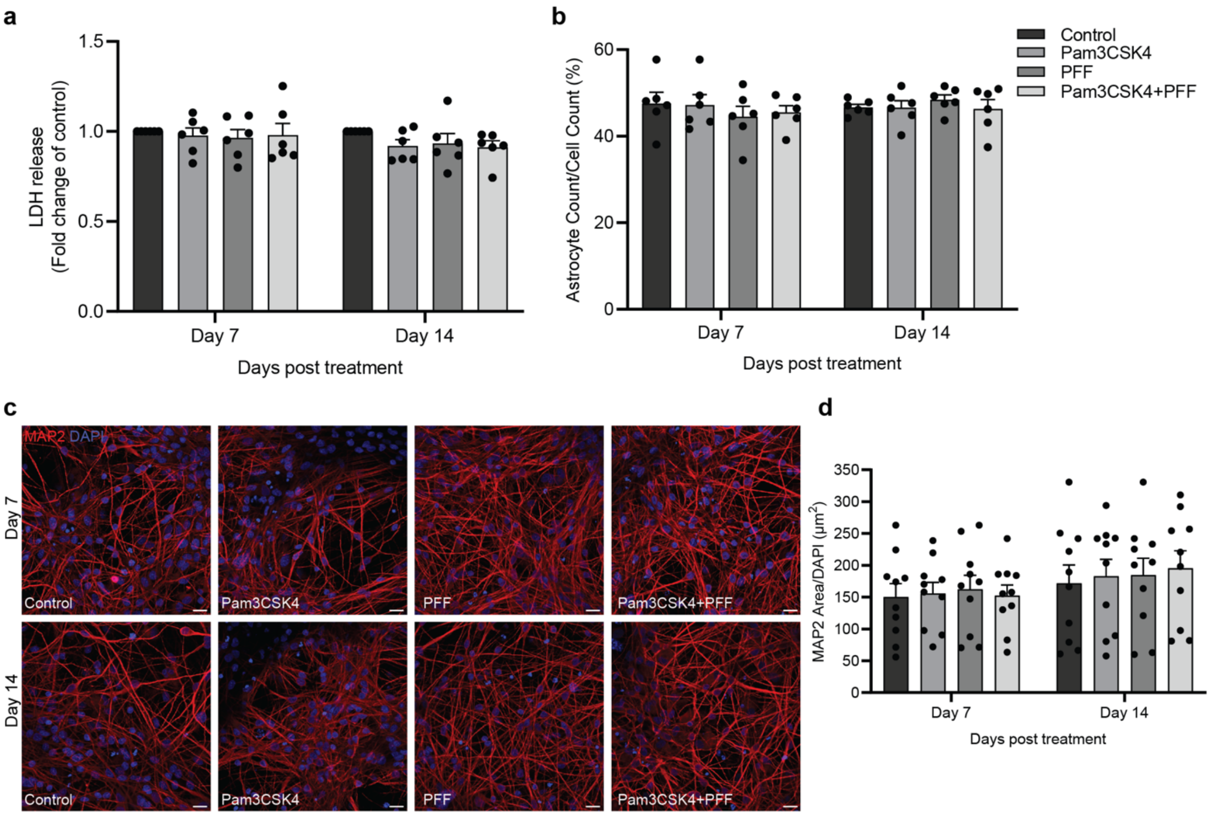

Figure S7: Treatment of Pam3CSK4 plus PFFs did not increase LDH released into the culture media or decrease MAP2 area. **a**, tissue culture media was collected at the time points indicated and measured for LDH release using CyTox96 assay kit. The graph shows LDH release expressed as the average fold change of the control ± S.E.M (n=6). **b**, graph shows number of S100β positive cells expressed as a percentage of the total cells, displayed as mean ± S.E.M (n=6). **c**, cells were fixed at the timepoints indicated and stained for MAP2 (*red*). Confocal images taken at 40X magnification, scale bar=20 µm. **d**, graph shows MAP2 area normalised to cell count as mean ± S.E.M. (n=10).

Table S1: List of antibodies used in this study.

Antibody Target	Species	Supplier (Cat#)	Application (Dilution)
α-synuclein	Mouse	BD Biosciences (610787)	ICC (1:300)<br>WB (1:1000)
Phosphorylated α-synuclein Serine 129	Mouse	BD Biosciences (825701)	ICC (1:250)
P62/SQSTMI	Mouse	Abcam (ab56416)	ICC (1:400)<br>WB (1:1000)
GFAP	Rat	ThermoFisher (13-300)	ICC (1:500)<br>Flow Cyt (1:300)
MAP2	Chicken	ThermoFisher (PA110005)	ICC (1:500)<br>Flow Cyt (1:500)
MAP2	Rabbit	Abcam (ab32454)	WB (1:1000)
TLR2	Rabbit	Abcam (ab191458)	WB (1:1000)
FOXA2	Mouse	Abnova (H00003170-M01)	ICC (1:400)
TH	Rabbit	ThermoFisher (OPA1-04050)	ICC (1:500)<br>Flow Cyt (1:300)
TH	Sheep	ThermoFisher (PA1-4679)	ICC (1:500)
S100β	Rabbit	Abcam (ab52642)	ICC (1:200)<br>WB (1:1000)
S100β	Mouse	Sigma (s2532)	ICC (1:500)
SerpinG1 (C1INH)	Rabbit	ThermoFisher (PA5-95890)	ICC (1:200)
Complement C3	Goat	ThermoFisher (PA1-29715)	ICC (1:500)
LAMP2A	Rabbit	Abcam (ab18528)	ICC (1:300)
PE anti-human CD282 (TLR2)	Mouse	BioLegend (309707)	Flow Cyt (1:20)
PSMB8	Rabbit	Abcam (ab3329)	ICC (1:200)
GBP2	Rabbit	ThermoFisher (PA5-112426)	ICC (1:250)
